# Analysis of Human Gait Using Hybrid EEG-fNIRS-Based BCI System: A Review

**DOI:** 10.3389/fnhum.2020.613254

**Published:** 2021-01-25

**Authors:** Haroon Khan, Noman Naseer, Anis Yazidi, Per Kristian Eide, Hafiz Wajahat Hassan, Peyman Mirtaheri

**Affiliations:** ^1^Department of Mechanical, Electronics and Chemical Engineering, OsloMet—Oslo Metropolitan University, Oslo, Norway; ^2^Department of Mechatronics and Biomedical Engineering, Air University, Islamabad, Pakistan; ^3^Department of Computer Science, OsloMet—Oslo Metropolitan University, Oslo, Norway; ^4^Department of Plastic and Reconstructive Surgery, Oslo University Hospital, Oslo, Norway; ^5^Department of Computer Science, Norwegian University of Science and Technology, Trondheim, Norway; ^6^Department of Neurosurgery, Oslo University Hospital, Oslo, Norway; ^7^Department of Biomedical Engineering, Michigan Technological University, Michigan, MI, United States

**Keywords:** gait, hybrid BCI, electroencephalogram, lower extremity, fNIRS

## Abstract

Human gait is a complex activity that requires high coordination between the central nervous system, the limb, and the musculoskeletal system. More research is needed to understand the latter coordination's complexity in designing better and more effective rehabilitation strategies for gait disorders. Electroencephalogram (EEG) and functional near-infrared spectroscopy (fNIRS) are among the most used technologies for monitoring brain activities due to portability, non-invasiveness, and relatively low cost compared to others. Fusing EEG and fNIRS is a well-known and established methodology proven to enhance brain–computer interface (BCI) performance in terms of classification accuracy, number of control commands, and response time. Although there has been significant research exploring hybrid BCI (hBCI) involving both EEG and fNIRS for different types of tasks and human activities, human gait remains still underinvestigated. In this article, we aim to shed light on the recent development in the analysis of human gait using a hybrid EEG-fNIRS-based BCI system. The current review has followed guidelines of preferred reporting items for systematic reviews and meta-Analyses (PRISMA) during the data collection and selection phase. In this review, we put a particular focus on the commonly used signal processing and machine learning algorithms, as well as survey the potential applications of gait analysis. We distill some of the critical findings of this survey as follows. First, hardware specifications and experimental paradigms should be carefully considered because of their direct impact on the quality of gait assessment. Second, since both modalities, EEG and fNIRS, are sensitive to motion artifacts, instrumental, and physiological noises, there is a quest for more robust and sophisticated signal processing algorithms. Third, hybrid temporal and spatial features, obtained by virtue of fusing EEG and fNIRS and associated with cortical activation, can help better identify the correlation between brain activation and gait. In conclusion, hBCI (EEG + fNIRS) system is not yet much explored for the lower limb due to its complexity compared to the higher limb. Existing BCI systems for gait monitoring tend to only focus on one modality. We foresee a vast potential in adopting hBCI in gait analysis. Imminent technical breakthroughs are expected using hybrid EEG-fNIRS-based BCI for gait to control assistive devices and Monitor neuro-plasticity in neuro-rehabilitation. However, although those hybrid systems perform well in a controlled experimental environment when it comes to adopting them as a certified medical device in real-life clinical applications, there is still a long way to go.

## 1. Introduction

Human gait is one of the most important human activities that require complex coordination between different brain regions, the musculoskeletal system, and the limb. Sensory inputs from the cerebral and sensory cortices activate the premotor and supplementary motor areas (SMA) of the cerebral cortex, where motor programs are created. It is believed that the cerebellum (Cunningham et al., 2010) is regulating the gait “error/correction” to coordinate proper movement by responding to abnormalities in posture (Takakusaki, 2013). BCI technologies perform differently during bipedal movements depending on different factors such as age, weight, and height (Samson et al., 2001; Mahlknecht et al., 2013; Elbaz et al., 2018). Gait disorders dramatically affect the quality of life and increase personal dependence on others (Pirker and Katzenschlager, 2017), which makes gait analysis an essential and timely research topic.

**Figure 1:**
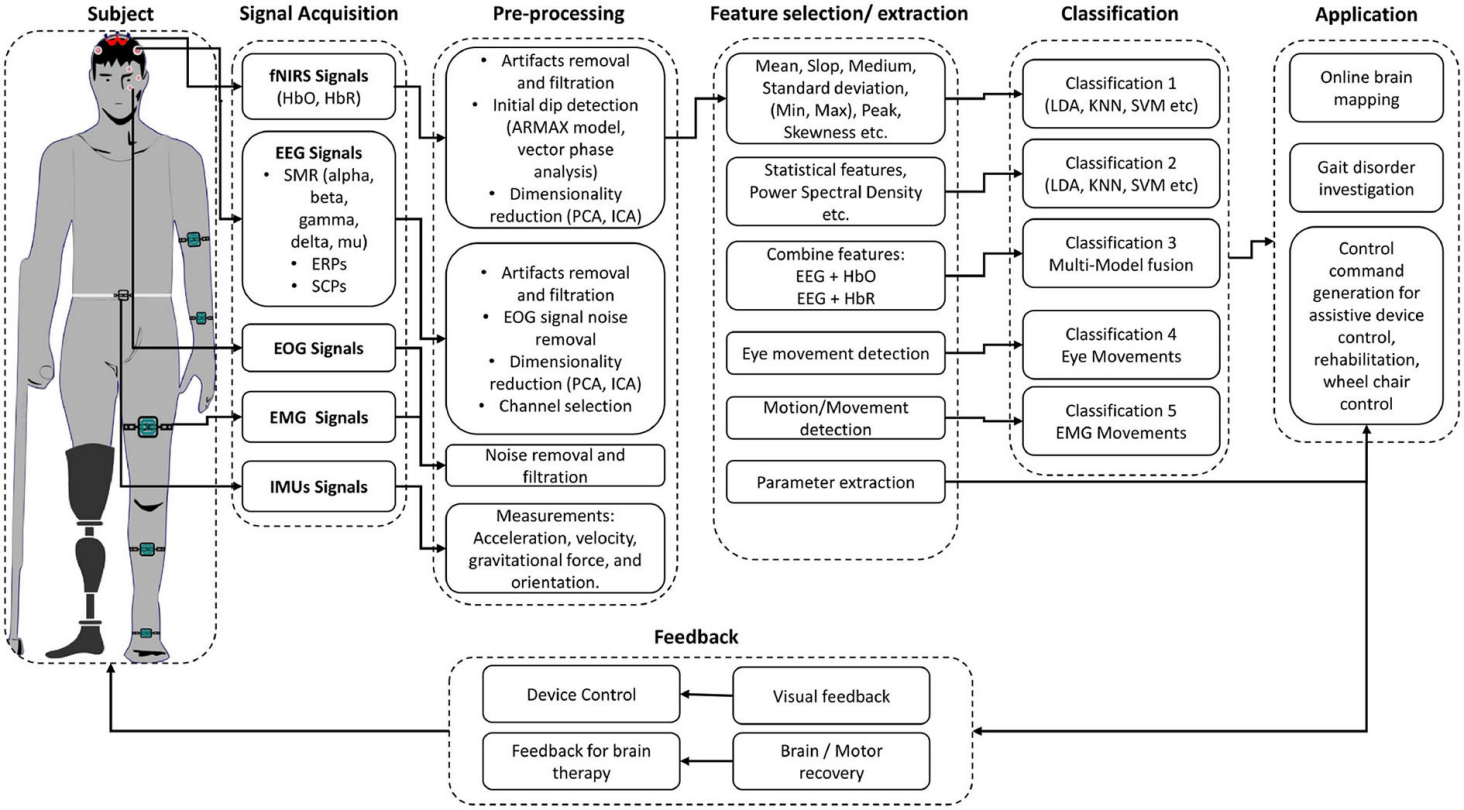
Hybrid BCI system block diagram for gait assessment.

In recent years, brain–computer interface (BCI) development has played a vital role in investigating musculoskeletal gait and brain dysfunction disorders. A typical BCI system consists of five main components, as shown in Figure 1: signal acquisition, pre-processing, feature extraction, classification, and the application interface (Naseer and Hong, 2015). BCI system can be used to restore the motor function by (1) feedback in real-time while performing motor imagery (MI) tasks; (2) representation of performed action in virtual reality; and (3) control of external devices causing actual movement using functional electrical stimulation (FES) (van Dokkum et al., 2015). BCI is also a promising tool for post-stroke rehabilitation. Indeed, BCI can be deployed to interface the neurofeedback for stroke patients and enhance cortical activation (Nowak et al., 2009; Mihara et al., 2012, 2013). Bamdad et al. (2015) review concluded that cognitive damage arising from brain injuries and neurological diseases could be reduced with the help of rehabilitation strategies involving BCI. BCI's performance depends on the type of neuro-system defect, the level of disability, the level of participation of the subject (Kübler and Birbaumer, 2008; Shanahan et al., 2017) as well as the aforementioned factors related age, weight, and height. For instance, when the degree of neuro-system defect increases, the user's ability to control the BCI system decreases. Similarly, the BCI's performance increases as the level of active participation increases. Shanahan et al. (2017) shows that the BCI system performs better in terms of accuracy and control for older people than children due to active participation and repetition of specific tasks leading to higher signal quality. It is worth mentioning that there are other wearable and non-wearable technologies used for gait and balance assessment (Shanahan et al., 2017; Singh et al., 2019), which do not involve brain signals. Examples of those wearable technologies include optical motion capture systems, instrumented walkway, and force platforms. Non-wearable technologies include pressure sensors and internal sensors (Shanahan et al., 2017). Although these wearable and non-wearable technologies help in understanding the information about the musculoskeletal systems and biomechanics of humans, they need to be used in conjunction with BCI technologies to acquire the brain activity and form a holistic understanding of the brain's neuronal correlation with the musculoskeletal system. Different brain signals such as functional magnetic resonance imaging (fMRI), magnetoencephalography (MEG), electroencephalogram (EEG), or functional near-infrared spectroscopy (fNIRS) are used in various gait applications for BCI applications. MEG and fMRI give excellent spatial and temporal resolution to study the under neuronal activities and cerebral blood flow changes for gait analysis. However, they are not portable modalities, which make them inappropriate for real-time experimentation of gait (Morshed and Khan, 2014).

When it comes to online BCI applications, non-invasive and portable brain signal modalities are convenient technologies for analyzing gait disorders. In this perspective, EEG and fNIRS are gaining popularity in the research community due to their non-invasive nature, easy use, and portability. EEG represents one of the earliest technologies for brain signal acquisition and has found various gait analysis applications. fNIRS is a relatively recent technology compared to EEG that was effective in capturing brain hemodynamics. It plays a vital role in various applications triggering a hemodynamical response such as motor rehabilitation (Khan R. A. et al., 2018). Pelicioni et al. (2019) reviewed fNIRS studies with a particular focus on prefrontal cortex (PFC) activation during walking and its effects on different age groups, type of disease, and secondary tasks performed during walking. The majority of those reviewed studies document an increase in PFC activation as a consequence of the increase of the complexity of the walking task in young, older people and patients with gait and balance disorder due to stroke, Parkinson's disease, cerebral palsy, head trauma, or other spinal cord injuries. Along those lines, increased PFC activation was documented in studies involving walks with dual tasks, where the secondary task can be arithmetic, verbal fluency, or alphabet reciting (Pelicioni et al., 2019). Different *single* brain signal modalities have advanced the research in different applications of BCI. However, the fusion of these brain signal modalities can provide complementary information to understand the brain signals better. It led to the emergence of a new sub-field within BCI called hybrid BCI (hBCI). hBCI combines two brains modalities or at least one brain modality with another non-brain signal acquisition modality (Pfurtscheller et al., 2010; Hong and Khan, 2017; Hong et al., 2018). Four factors are essential for any hBCI system: (1) signals should be acquired directly from brain activity; (2) among the brain signals, at least one signal must be intentionally controlled; (3) a signal must be processed in real-time to develop communication between the brain and computer; and (4) feedback control must be provided to evaluates the outcome.

Fused EEG-fNIRS showed its significance in the various cognitive investigation such as Li et al. (2019) studied cognitive deficits in Alzheimer's patients, and concluded that fused EEG-fNIRS could help better in understanding the spatiotemporal dynamics of the brain activation. Integration of EEG-fNIRS provides complementary properties of high temporal and moderate spatial resolution Li et al. (2020c). The fusion of different bio-signals in hBCI permits to enhance classification accuracy (Cicalese et al., 2020), increase the number of control commands and reduce the signal processing time (Hong and Khan, 2017). When it comes to investigating gait problems and developing real-time BCI-based control, hBCI is attracting more and more attention. Conventionally, co-located modalities monitoring the same brain regions help increase classification accuracy by 10–20% comparative to individual modalities (Hong and Khan, 2017; Cicalese et al., 2020). Conversely, placing the modalities over different regions helps to enhance the number of control commands (Hong and Khan, 2017). From our literature survey focusing on hBCI for gait analysis, we conclude that individual EEG and fNIRS-based BCI are commonly used separately with the exception of one study that resorts to a fused EEG-fNIRS for tetraplegia (Blokland et al., 2013). The fusion of EEG and fNIRS is discussed in detail in section 3.1.3. Table 1 shows the possible advantages of combining these brain signal modalities (EEG and/or fNIRS) with other modalities for different applications in general, meaning not necessarily gait applications. Thus, the potential of hBCI is yet to be explored for gait applications. The review will provide insight into hybrid EEG-fNIRS BCI systems for investigating gait while focusing on the development made in each component of the BCI system.

**Table 1:** Combination of the brain and non-brain signals modalities.

**References**	**Modalities combination**	**Signal fusion**	**Benefits**
Li et al. (2016) and Hong and Khan (2017)	EEG + EOG	Electrophysiological + Ocular Movements	1. To enhance accuracy and number of control commands. 2. To detect the motion artifacts due to ocular movements.
Bulea et al. (2014); Sburlea et al. (2015); Gui et al. (2017); Hong and Khan (2017), and Liu D. et al. (2017)	EEG + EMG	Electrophysiological + Electromyography	1. To increase the accuracy of the BCI system. 2. This combination could help to make comprehensive neural BCI and minimize the delay between movement detection and execution. 3. It can help to enhance the active participation of patients in gait rehabilitation. 4. EMG is usually used with EEG to detect the actual muscle movement to ensure BCI's smooth operation.
Fazli et al. (2012); Blokland et al. (2013); Khan et al. (2014); Hong and Khan (2017), and Cicalese et al. (2020)	EEG + fNIRS	Electrophysiological + Hemodynamics	1. To enhance classification accuracy. 2. To increase the number of control commands.
Tobar et al. (2018)	EEG + fMRI + EMG	Electrophysiological + Hemodynamics + Electromyography	1. fMRI is used to locate the brain activation area, EEG is used to record to cortical activity, while EMG is used to confirm motor task execution.
Zhang et al. (2010) and Liu et al. (2018)	EEG + EOG + EMG	Electrophysiological + Ocular Movements + Electromyography	1. The actual movement onset was extracted from surface EMG, and the motor intention was detected from EEG. EOG is used to detect ocular movement artifacts. 2. To increase the number of control commands.
Salazar-Varas et al. (2015); Hortal et al. (2016); Gui et al. (2017), and Elvira et al. (2019)	EEG + IMUs	Electrophysiological	1. IMUs are used along with EEG to detect actual body movements.

In addition, the review will elaborate on the potential of EEG-fNIRS-based hBCI for gait analysis. The remainder of this article is structured as follows: review methodology is provided in section 2, hybrid EEG-fNIRS-based BCI is discussed in section 3, section 4 provides discussion around prospect, and finally, section 5 presents conclusive remarks on hybrid EEG-fNIRS for gait analysis.

## 2. Review Methodology

Our review paper follows preferred reporting items for systematic reviews and meta-analyses (PRISMA) guidelines to examine EEG and fNIRS-based BCI systems for gait (Moher et al., 2009).

### 2.1. Search Strategy

To ensure the relevance of the articles different keywords was structured, as shown in Table 2. Moreover, articles from other sources by manual search and reference articles from included studies were also included.

**Table 2:** Search string used for a literature survey.

**Combination of keywords**	
AND	fNIRS OR functional near-infrared spectroscopy OR EEG OR Electroencephalography OR Bio-signal OR Brain signal OR Neuro-imaging OR Optical brain imaging
AND	Gait OR Walking OR Balance OR Sway OR Bio-mechanics OR Bio-mechanics OR Posture OR Postural control
AND	Neurological disorders OR Neural disease OR Neural disorders OR Stroke OR Neuro-rehabilitation OR Cognition OR Motor-cognitive OR Gait disorders
AND	Brain-Computer Interface OR BCI OR Human-machine interface OR Brain-machine interface

### 2.2. Inclusion and Exclusion Criteria

**Figure 2:**
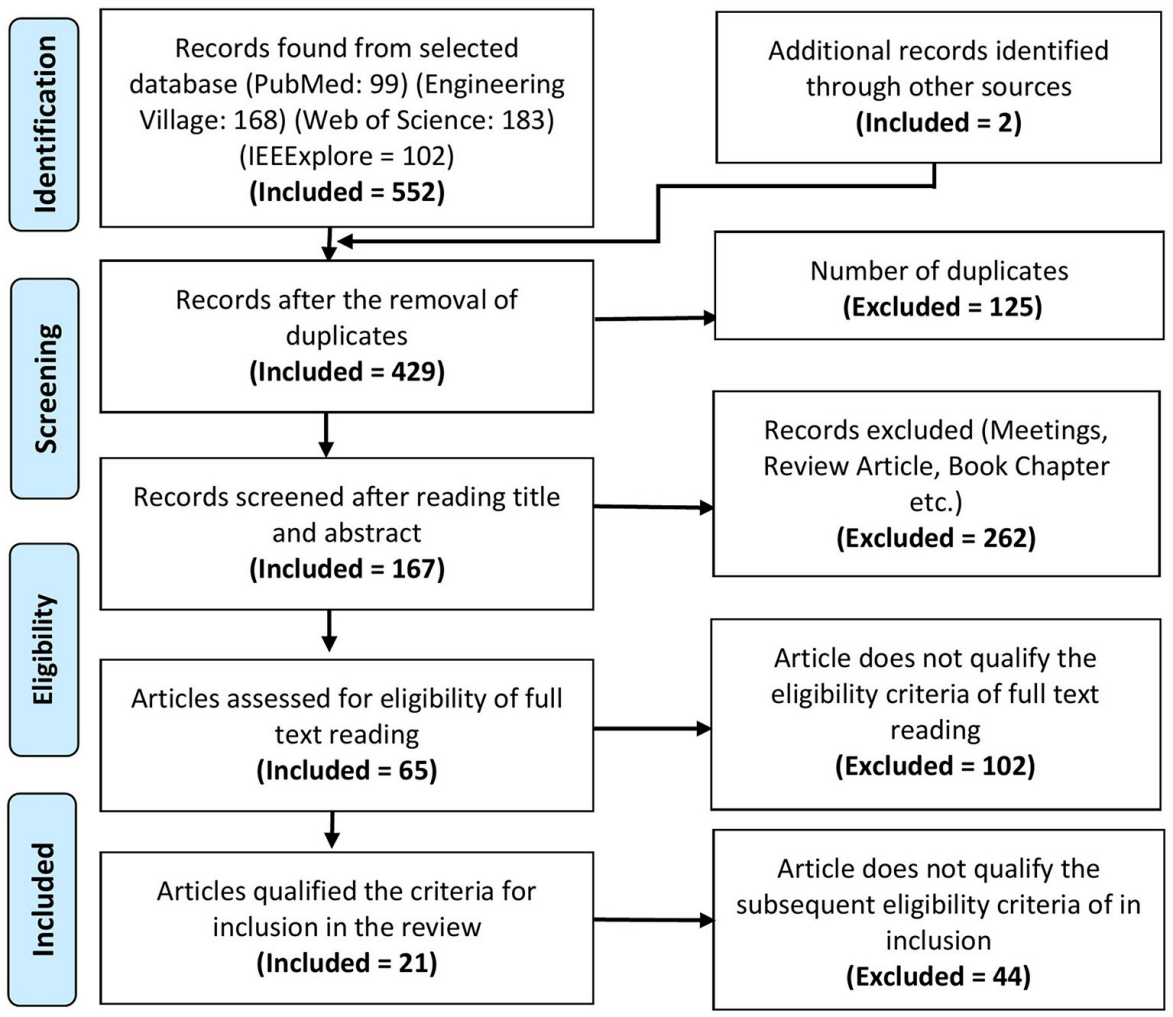
PRISMA flowchart of the article selection.

A total of 552 articles were collected from PubMed, Engineering village, Web of science, and IEEExplore databases. The PRISMA flowchart shows the complete selection procedure in Figure 2. EndNote and Mendeley were used in processing, screening, and filtrating of articles. Manual verification is also done to verify the process. The exclusion criteria from the first phase of screening title of articles were screened to select the articles for abstract reading having following exclusion criteria: (1) manuscripts which are not broadly in line with the topic, i.e., gait; (2) articles such as book sections, review papers, lecture notes, and meeting minutes were excluded; (3) brain signals used for other than gait applications; and (4) articles with the question on their authentication. In the next phase of the screening stage, paper abstracts were read out to consider the articles for full-text reading that satisfy the following criteria: (1) articles focusing on gait, balance, or neurological disease; (2) manuscripts describing the whole BCI system; (3) at least used one type of non-invasive and portable brain signal modality; (4) experimentation with only motor imaginary (MI) tasks are not included; (5) experiments conducted on animals; and (6) articles related to upper limb prosthesis and rehabilitation were excluded. In the eligibility stage, manuscripts that have contribution or detailed discussion in all three main components of BCI, i.e., signal acquisition, signal processing, and application of control signals, are selected. Furthermore, the following criteria for the inclusion of articles in the review that are considered: (1) articles focusing on the use of EEG or fNIRS or hBCI are included; (2) manuscripts with details of experimental description are considered; (3) description of signal filtration methods and machine learning algorithm explained; and (4) details of application of control signal or interface application.

### 2.3. Data Extraction

The following information is mainly extracted from the manuscripts that passed the eligibility stage: (1) Author and year of publication; (2) Aim of study; (3) Assessment methodology; (4) Signal processing; and (5) Main findings. Furthermore, other relevant information related to signal acquisition and processing is presented in relevant sections.

## 3. Hybrid EEG-fNIRS-Based BCI

Section 3 gives a brief overview of the existing signal acquisition methods (section 3.1), pre-processing methods (section 3.2), feature extraction and selection methods (section 3.3), classification algorithms (section 3.4), and existing gait application in (section 3.5). The conclusion and finding of selected articles in this review article are presented in Table 3. Table 4 summarized the signal processing methods applied in the selected studies. The Commonly used BCI components used in the manuscripts are discussed in the upcoming and relevant sections.

**Table 3:** Summary table of reviewed articles.

**References**	**Aim of study**	**Assessment task**	**Main findings**
Blokland et al. (2013)	The study aimed to investigate the feasibility of using a combination of EEG-fNIRS in tetraplegia patients. Secondly, it aims to test the feasibility of motor execution instead of MI as a brain switch control task.	Subjects performed six sequential movement tasks; each task consists of six trials with visual aids. Each trial lasted for 15 s. Three different tasks were completed, i.e., “rest” (do nothing), “movement” (fingers and thumb tapping continuously), and “imagined movement” (imagine tapping of fingers and thumb continuously).	EEG-fNIRS system proved to be beneficial for users who lack sufficient control of current EEG-based brain switches. The average classification performance was 87% for the motor attempt and 79% for MI in tetraplegia patients.
Do et al. (2013)	The research aims to investigate the feasibility of a BCI system for lower extremity prosthesis patients. EEG, EMG, and gyroscope are used along with commercial robotic gait orthosis devices to investigate BCI's feasibility for motor disability of SCIP.	One orthosis and one healthy patient were considered in this study with 30-s idling and ten mint kinematic motor imagery tasks, respectively, and the additional walking task at a 2 km/hr speed for a healthy subject.	The offline accuracy for healthy and SCIP was 94.8 ± 0.8% and 77.8 ± 2.0% receptively. The BCI-RoGO system helped to regain brain driven basic ambulation with less training time. The results are quite convenient to use BCI based systems for rehabilitation and restoring overground walking.
Rea et al. (2014)	The study aimed to assess the measurement and classification of hemodynamic signals associated with lower limb motor movements for chronic stroke and its usage for future fNIRS-BCI rehabilitation applications of the lower limb.	The experimental paradigm consisted of two sessions with eleven left and right hip of movement preparation (9–11 s) followed by movement execution (left/right hip) (3 s), and then in between rest (15–25 s) performed in a pseudo-randomized order	Single-trial analysis indicated that specific hemodynamic changes associated with the left and right hip movement preparation could be measured with fNIRS with classification accuracies of 73 and 89%, respectively. These findings encourage further investigations of fNIRS suitability for BCI applications in rehabilitating patients with lower limb motor impairment after stroke.
Bulea et al. (2014)	In this study, EEG delta band is used to investigate the intention before movement execution to differentiate between three different classes, i.e., rising from sitting position to standing, lowering from standing position to sitting, and standing or sitting quietly.	Ten healthy adults participated in the experiment of performing three different tasks during two trials. One trail was self-paced, and the other is audio-triggered. Each trial has ten alternate sit to stand and stand to sit transition. Standing or sitting in the third task, which was random of 3–10 s.	Classification accuracy of 78% is achieved using features extracted from pre-movement epochs with no post-processing required and minimizing the classification delay. Result suggests that the primary motor cortex (M1) contains more discriminative information for standing and sitting intention when movements are self-initiated compared to cue.
Salazar-Varas et al. (2015)	The study focuses on the early detection of subsequent alertness after the obstacle's sudden appearance using EEG signals. This work's final application is to generate the STOP control command for exoskeleton control in obstacle detection.	Different responses regarding obstacle appearance were detected, such as reaction (stop walking after the appearance of an obstacle), delayed reaction (continuous few steps after the appearance of an obstacle), no reaction (ignore the obstacle), and free reaction (subject freely decides when to stop). A run consists of 180 s of Reaction condition, 240 s of Delayed reaction condition, 180 s of No reaction condition, and 120 s of Free reaction condition.	The results obtained for the majority of the subjects showed that polynomial coefficients achieved have the lowest false positive rate and a high true positive rate (mean accuracy of 79.5%), which shows the feasibility to detect the obstacle before the subject reacts. The slope feature offers acceptable performance for classification.
Sburlea et al. (2015)	This study focuses on the design of an EEG-based decoder that combines temporal and spectral features to detect early movement states in stroke patients. The study also summarizes the patient's intrinsic motivation when performing gait rehabilitation.	Each trail consists of two-part, i.e., relaxation and movement. Relaxation time was 10 s, followed by a beep to start a movement which of patient dependent time length. The single trial consists of 20 repetitions. In a single week, the subject performs three trials that form 100 relaxation and movements.	It is concluded that using a decoder that combines temporal and spectral features can detect pre-movement state with an accuracy of 64% in a range between 18 and 85.2%, with the chance level at 4% in stroke patients. Furthermore, it was found a significantly strong positive correlation (*r* = 0.561, *p* = 0.048) between the motivation of the patients to perform the rehabilitation related task and the accuracy of the BCI detectors of their intention to walk.
Lopez-Larraz et al. (2016)	This study proposes an EEG based closed-loop BMI system for control of an ambulatory exoskeleton for gait rehabilitation of SCIP without any balancing and support.	The BMI session is performed consisting of two sessions; one is screening blocks, and the other is closed feedback blocks. The subjects performed 3–4 screening blocks each of 20 trials to calibrate the BMI decoder. In closed-loop feedback, the block consists of four intervals, i.e., rest, preparation, movement attempt, and movement.	Three out of four patients performed at least one successful BMI session with an average performance of 77.61 ± 14.72%. All the patients showed low exertion and fatigue levels during the experiments, which validate the closed-loop BMI system for gait rehabilitation.
Hortal et al. (2016)	In this work, a BMI based on the event-related desynchronization and event-related synchronization phenomena are developed to control a lower limb exoskeleton. The BMI can detect the gait starting and stopping pattern.	In each of these sessions, the subject performed several runs (8 or 10). Each run consists of 10 repetitions. Each run starts with 10 s of relaxation time, the 10 s of the walking task, and 5-s rest at the end.	In both preliminary optimization analysis and real-time tests, the results obtained are very similar. The true positive rates are 54.8 and 56.1%, respectively. Regarding the false positive per minute, the values are also very similar, decreasing from 2.66 in preliminary tests to 1.90 in real-time. Finally, the average latencies in detecting the movement intentions are 794 and 798 ms and preliminary and real-time tests. The existing system has the potential to use in real-time BMI for gait rehabilitation.
Li et al. (2016)	In this research, EEG signals are recorded to identify two different types of gaits, like movements and phase synchronization between brain regions.	The experimental procedure started with a resting period of 1 min; subjects were lying in a vertical position at an angle of about 55–75 degrees. Afterward, the subjects were instructed to perform automatic gait-like stepping movements (25–30 steps per min).	Our results suggested that brain activities were altered in different frequency bands after SCIP, which supported diverse neural networks with different resonance-like frequencies in the brain. In attempted/active movement, spatial function, and multi-modal integration with somatosensory information were crucial aspects of PMC, the function which needs to be considered separately in different EEG bands.
Gui et al. (2017)	This work aims to develop a lower-limb robotic exoskeleton with multiple gait patterns that can be controlled by users' intention. For subject's active participation enhancement, a multi-modal HRI system is established, which includes cognitive HRI and physical HRI. The BCI was used to identify four typical locomotion modes: stop, regular walk, acceleration, and deceleration.	A central pattern generator (CPG) is created to create joint trajectories. The relative state variables of the locomotion mode, i.e., amplitude, frequency, and offset, were transferred to the central pattern generator (CPG) for command generation. In this way, the rehabilitation system is expected to achieve desired assistive gait patterns regulated by EMG based pHRI and EEG based cHRI.	EEG and EMG based HRI to enhance the active participation of patients for gait rehabilitation has been designed. According to the user's voluntary intention, the state variables of CPG are changed through EEG-based cHRI and EMG-based pHRI. The results show that the proposed system incorporates voluntary and active movement consciousness of healthy subjects and stroke patients.
Liu D. et al. (2017)	This study describes the impact of different feedback modalities on the performance of an EEG-based BMI that decodes MI of leg flexion and extension. Firstly, an online decoder is built to classify MI, secondly, analyze the effect of visual and proprioceptive feedback on BMI performance, and discriminate features and brain modulations among and within-subjects in this paradigm.	Each participant performs three sessions with a break of a week in between. Each session was composed of 5 runs of approximately 10 min with a resting time of 5 min in between. Each run consisted of 60 trials with extension and flexion cue balanced and randomized inside.	The results suggest that proprioceptive feedback has an advantage over visual feedback. In real-time classification, the average accuracy was 62.33 ± 4.95 and 63.89 ± 6.41% for the two online sessions. The study reported a closed-loop brain-controlled gait trainer as a proof of concept for neuro-rehabilitation devices.
Zhang et al. (2017)	This research's primary goal is to classify different gait intentions, i.e., stop, walk, turn left, and turn right from the EEG signals. The other objective is to identify brain areas employed to classify different gait movements in both healthy and SCIP.	The first session consists of four different tasks, i.e., walking forward, turning left, turning right, and stopping. The second was walking and stop with multiple sessions and at least ten walks to stop and stop to walk transitions.	Using MKL and optimal kernel weights simultaneously prove the feasibility of classifying internal gait states from the EEG signals. It also helped to identify and learn through a group of features from relatively active brain areas.
Contreras-Vidal et al. (2018)	The study investigates the neural decoding and finds out the relationship between gait kinematics corresponding with neural changes while performing overground gait therapy for chronic stroke patients.	Six Chronic post-stroke hemiparesis patients participated in the experiment of 12 sessions for 4 weeks. H2 robot-assisted exoskeleton was used for training.	A significant relationship between decoding accuracy, total steps, and walking speed was found in the study. The synchronization of EEG signals from the brain, kinematics, and dynamics feedback from the exoskeleton can promote brain reorganization due to motor learning expected because of activity-dependent brain plasticity.
Tobar et al. (2018)	The study's objective is to decode cortical activity using EEG signals for ankle flexion and extension at two different force levels in both legs. fMRI is used to locate the brain's anatomical areas, contributing to motor execution and ankle movements.	Eight participants of age mean 29.67 ± 8.81 participated in the experiment. Different experiments on EEG and fMRI were conducted on different days. A total of 8 active tasks were performed in both experiments.	Classification accuracies of 65.64 and 22.19% for estimated current sources and EEG sensor signals with (11.11%) above chance level were obtained. fMRI recording helped to identify the specific areas to generate control commands.
Liu et al. (2018)	This research aims to decode the plantar flexion movement intention using continuous classification and asynchronous detection in a gait training paradigm for self-paced gait using movement-related cortical potentials.	Each experimental run consists of 3 consecutive tasks. Participants were asked to relax and fix their eyes on the cross in the monitor's center for 1 min for calibration. In the second task, participants performed self-paced plantar flexion five runs of 10 min, with a rest period of 3 min in between. Each run consisted of 60 trials, with left and right directional cues randomized and balanced inside.	With the proposed movement detection method, a higher true positive rate, lower false positives, and comparable latencies are achieved compared to the existing online detection methods. No significant differences were observed b/w left and right legs regarding neural signatures of movement and classification performance.
Hedian et al. (2018)	The study aims for intention detection and classification of fNIRS signals using two variables for motion intention detection, i.e., step length and walking speed. It aims to classify between three different states, i.e., small steps with low speed, small steps with mid-speed, and mid-step with slow speed.	All the subjects were asked to walk at a distance of 4.4 m with three walking states, i.e., the gait of small-step with low-speed, small-step with mid-speed, and midstep with low-speed. All three states were repeated twice with the rest of 30 s in between and backward process tasks to move back to resting position.	In this study, fNIRS-based automatic gait intention detection (walking speed and step size), with the classification accuracy of 78.79%, is achieved. The results confirm the use of fNIRS based BCI for rehabilitation.
Khan R. A. et al. (2018)	The work introduces a novel fNIRS-based BCI system that can be used to control prosthetic leg and further utilize it to rehabilitate patients with gait disorders. The study focuses on optimal feature extraction and feature extraction to enhance the classification accuracy using different MLA.	The experimental paradigm consists of a baseline rest of 30 s before the start of the experiment. Subjects were asked to walk on a treadmill for 10-s followed by a rest of 20 s in a single trail. Each subject was asked to perform ten trials with the rest of 30 s between the trials.	The classification accuracies obtained for SVM was higher (75%) relative to other classifier using the hrf were significantly higher (*p* <0.01). Subject-wise accuracy was 77.5, 72.5, 68.3, 74.2, 73.3, 80.8, 65, 76.7, and 86.7% for the nine subjects, respectively.
Costa-Garciacutea et al. (2019)	The study aims to perform online classification of EEG signals, minimize the possible artifacts during gait, reduce classification time, and enhance the system's online accuracy.	Dual tasks were performed, with the primary task was asking the participant to walk on a treadmill with a speed of 2 km/h, while the secondary task was designed to change the participants' attention level. The secondary task was composed of three 30 s trials having mental arithmetics followed by a regular walk and then walking, followed by markers that correspond to low, medium, and high attention levels.	The noisy electrode was removed using MVT, instant kurtosis, and spectral power. The average success rate was enhanced to 69% for healthy subjects while 57% for SCIP using LDA.
Elvira et al. (2019)	The study aim was the detection of an unexpected obstacle during normal gait using EEG signals. The study also improves accuracy and reduces the false positive rate in comparison to the previous studies by using IMUs and improvement in feature extraction.	Each subject performed ten trials, which last for 2 min. Each subject is asked to complete a walk at a constant velocity of 2 km/h. During this time, the laser line is projected randomly for one second. The interval between two successive stimuli varies between 6 and 9 s–pa total of 12 and 14 lasers appear on each trial. The subject is instructed to suddenly stop when the laser is visualized and then resume gait afterwards.	The pseudo-online results of the BMI for detecting the appearance of obstacles, with an average percentage of 63.9% of accuracy and 2.6 false positives per minute, showed a significant improvement compared to previous studies.
Li et al. (2020a)	The study focuses on fNIRS-BCI for dynamic regulation of two different motion intention states in a realistic environment and detecting movement intension during self-regulated states instead of a resting state. It uses the inter-subject BCI instead of within-subject BCI with improvements in MLA to enhance inter-subject BCI performance.	Subjects were asked to walk at four different self-adapted states, i.e., speed increase, speed reduction, step increase, and step reduction. At the end of every gait, state subjects stop and take a rest for a minimum of 30 s. Every walk state is repeated twice during a single run of the experiment.	GBDT performed well in detecting the onset intention. The 2-layer-GA-SVM model increased the average accuracy of four types of intention from 70.6 to 84.4% (*p* = 0.005) from the single GA-SVM model. It uses inter-subject BCI instead of within-subject BCI with improvement in MLA.
Li et al. (2020b)	The study aimed to generate control command from fNIRS signals obtained from the brain to control assistive devices using walking intensions.	Each trail consists of two-part, i.e., relaxation and movement. Relaxation time was 10 s, followed by a beep to start a movement which of patient dependent time length. The single trial consists of 20 repetitions. In a single week, the subject performs three trials that form 100 relaxation and movements.	TKE is used to extract the features and GBDT to detect the walking intention at each sampling point. The walking model recognition results proved that it is feasible to detect the self-paced intention based on NIRS technology.

*BMI, Brain-Machine Interface; CPG, Central Pattern Generator; EEG, Electroencephalography; EMG, Electromyography; fNIRS, Functional Near-Infrared Spectroscopy*.

*GBDT, Gradient Boosting Decision Tree; HRI, Human-Robot Interaction; LDA, Linear Discriminant Analysis; MI, Motor Imaginary; MKL, Multiple Kernel Learning*.

*MLA, Machine Learning Algorithms; MVT, Maximum Visual Threshold; PMC, Primary Motor Cortex; SCIP, Spinal Cord Injured Patients; TKE, Teager-Kaiser Energy*.

**Table 4:** Summery of signal processing methods.

**References**	**Summary of signal processing methods**
Blokland et al. (2013)	Pre-processing: fNIRS: High pass filters (HPF) (0.01 Hz), low pass filter (LPF) (0.2 Hz), EEG: down sampling to 256 Hz Features: For fNIRS HbO, HbR, For EEG power spectral features Classifier: *L*_2_−regularized linear logistic regression classifier
Do et al. (2013)	Pre-processing: EEG prediction model, approximate information discriminant analysis Features: Spatio-spectral features Classifier: Classwise principal component analysis (PCA), linear Bayesian classifier
Rea et al. (2014)	Pre-processing: Wavelet-minimum description length algorithm, Gaussian Low pass filter Features: Mean changes in HbT concentration in PMC and PPC were used as discriminatory features Classifier: Linear discriminant analysis (LDA)
Bulea et al. (2014)	Pre-processing: Butterworth filter, ASR filter, band-pass filter (BPF), Low pass filter (LPF) Feature extraction method (FEM): Teager-Kaiser energy (TKE) Classifier: PCA, LDA, GA, LFDA-GMM
Salazar-Varas et al. (2015)	Pre-processing: Common average reference (CAR) FEM: Common spatial pattern Classifier: LDA
Sburlea et al. (2015)	Pre-processing: Software's EEGLAB and FastICA were used for artifacts sLORETA FEM: Features were extracted using MRCP and event-related (de)synchronization Classifier: Sparse linear discriminant analysis
Lopez-Larraz et al. (2016)	Pre-processing: Z-score based automatized artifacts removal FEM: Sliding window method (SWM), Sparse discriminant analysis
Hortal et al. (2016)	Pre-processing: LPF, 8*th* Order Butterworth filter FEM: Fast Fourier Transform is used to obtain spectral power and then further used for feature extraction Classifier: Support Vector Machine (SVM)
Li et al. (2016)	Pre-processing: Regression analysis algorithm FEM: LORETA method is used to measure cortical activity, fourteen regions of interest were obtained from the segmentation of Brodmann areas
Gui et al. (2017)	Pre-processing: 4*th* order Butterworth filter Features: Four locomotion modes to be identified by SSVEP: (ST, 12.5 Hz), (NW, 8.33 Hz), (AC, 7.5 Hz), and (DE, 6.82 Hz) Classifier: LDA
Liu D. et al. (2017)	Pre-processing: CAR Classifier: Random Forest
Zhang et al. (2017)	Pre-processing: ASR filter, 2*nd* Butterworth filter and standardized (z-score) Features: Multiple learning algorithm (MKL) Classifier: Kernel-based learning (KBL)
Contreras-Vidal et al. (2018)	Pre-processing: LPF (3 Hz), ASR filter, Butterworth filter, Kalman filter Classifier: PCA
Tobar et al. (2018)	Pre-processing: BPF, down sampling FEM: Variational Bayesian Multimodal Features: Down sampled epochs Classifier: Sparse logistic regression
Liu et al. (2018)	Pre-processing: : 6*th* Order Butterworth filter, CAR, Weighted average (WAVG) filter FEM: TKE operator Classifier: Random Forest
Hedian et al. (2018)	Pre-processing: Mathematical morphology filter (MMF) FEM: Power spectrum analysis Features: HbO, HbR, and HbT Classifier: SVM
Khan R. A. et al. (2018)	Pre-processing: Kalman, Wiener, Gaussian, hemodynamic response filter, band-pass, finite impulse response FEM: Spatial Averaging Features: Signal Mean, signal slope, signal variance, slope kurtosis, signal peak, and signal skewness Classifier: k-Nearest neighbor (KNN), Quadratic discriminant analysis (QDA), LDA, Naïve Bayes, SVM
Costa-Garciacutea et al. (2019)	Pre-processing: Maximum value threshold FEM: Maximum entropy method Features: Features contain information about synchronizations and desynchronization Classifier: LDA
Elvira et al. (2019)	Pre-processing: BPF, signals with a standard deviation greater than 40 microV have been removed, Channels with artifact are manually removaed Classifier: LDA
Li et al. (2020a)	Pre-processing: 2*nd* order low pass Chebyshev filter, MMF FEM: Genetic algorithm (GA), average over regions of interest by entropy weight method, time-domain, and correlation analysis feature extraction Features: Mean, standard deviation, coefficient of variation, energy, range, skewness, kurtosis, peak, and Hjorth parameters Classifier: Light gradient boost decision tree, two layer-GA-SVM, PCA
Li et al. (2020b)	Pre-processing: Chebyshev bandpass filter, z-score FEM: TKE Features: HbO, HbR, and HbT Classifier: Gradient boosting decision tree model

### 3.1. Signal Acquisition

The first important step in hBCI is the acquisition of brain signals. For hBCI related to gait disorders, brain signals are usually acquired from the motor cortex, which corresponds to MI and motor execution (ME) (Li et al., 2016; Mrachacz-Kersting et al., 2017; Rodriguez-Ugarte et al., 2018). The symmetry of signal acquisition hardware used for the selected articles is shown in Table 5. This section introduces EEG and fNIRS brain signal modalities and their fusion with other brain and non-brain signal related modalities.

**Table 5:** Hardware and software description used for signals acquisition for gait applications.

**References**	**Brain** **signals**	**Brain signal** **device**	**Other** **signals**	**Signal** **amplifier**	**No. of ** **channels**	**Sampling ** **frequency** **(Hz)**	**IPS**	**Brain area**	**Robotic system/** **softwares/** **other systems used**
Blokland et al. (2013)	EEG + fNIRS	EEG Porti system (TMSi), fNIRS (OxymonMK III, Artinis)			8 EEG, 3 fNIRS (2 E & 1 D)	2,048 (EEG), 250 (fNIRS)	10-20	C3-C6, FC3-FC4, CP3-CP4 for EEG, C3-C4 for fNIRS	
Do et al. (2013)	EEG		2 EMG electrode and Gyroscope	NeXus-32 bio amplifier, Mind Media	64	256		Whole scalp	Robot operated Gait Orthosis (RoGO), Lokomat
Rea et al. (2014)	fNIRS	ETG-4000, Hitachi Medical Systems GmbH	2 EMG, Brain Products GmbH	QuickAmp amplifier (Brain Products GmbH)	48	10	10-20	Primary & secondary motor areas, PMC, PMA, SFC, and somatosensory areas	NIRS-SPM and SPM5 by Welcome Trust Center for Neuroimaging
Bulea et al. (2014)	EEG	Brain Products	EMG sensor, Biometrics Ltd		64	1,000	10-20	Whole scalp	MATLAB, EEGLAB Software (Plugin)
Salazar-Varas et al. (2015)	EEG	BADYBird electrode	Several IMUs	USBamp, g.Tec company	32	1,200	10-10	Fz, Cz, FCz, FC(1,3,5,6), C1-C6, FC2, CPz, CP5-CP6, P1-P4, Pz, POz, PO7, PO3, PO8, and PO4	MATLAB and MATLAB with API by gUSBamp
Sburlea et al. (2015)	EEG	EEG caps, TMSi, Enschede	2 EMG bipolar Ag/AgCl Electrodes	TMSi Refa amplifier	30	256	10-10	Fp1,Fpz„Fz,Fp2,F7-F8,F3-F4, FC1-FC2,FC5-FC6, C3-C4, Cz, T7-T8, CP5, CP1-CP2, CP6, P7-P8, P3-P4, Pz, POz, O1, Oz, O2.	EEG lab 13.2.2 FastICA
Lopez-Larraz et al. (2016)	EEG	EEG headset, Tec system Graz		Amplifier used	32	256	10-10	AFz, FC3-FC4, FCz, C1-C6, Cz, CP1-CP4 CPz, FP1, FP2, F7-F8, F3-F4, Fz, T7-T8, P7-P8, P3-P4, Pz, O1, and O2	6 DOF wearable lower limb orthosis exoskeleton
Hortal et al. (2016)	EEG	g.LADYbird	IMUs, Tech MCS, Technaid S.L.	g.USBamp	9	1,200	10/10	Fz, FC1-FC2, C3-C4, Cz, CP1-CP2 and Pz	MATLAB with API by gUSBamp, actiCAP for electrode placement
Li et al. (2016)	EEG	SynAmps2 Quik-Cap, Compumedics Neuroscan	4 EOG Electrode		64	1,000	10-20	PMC	Erigo dynamic tilt table (Hocoma AG), Curry software (Compumedics Ltd)
Gui et al. (2017)	EEG	EMOTIVE EPOC, Emotive Systems	EMG signals, Biometrics			128			4 DOF in the sagittal plane custom made prototype of leg exoskeleton.
Liu D. et al. (2017)	EEG	BioSmei Active System	4 EMG Electrodes		32	2,048	10/20	F3-F4, FC1-FC2, FC5-FC6, CP1,CP5-CP6, P3-P4, Pz, CP2, C3-C4, FZ, and Cz	Gait Trainer LegoPress, Python MNE, Matlab R2015b with EEGLAB 13.5.4
Zhang et al. (2017)	EEG			Two amplifiers, Brain Products GmbH	64		10-20		The Wearable exoskeleton, REX Bionics Ltd
Contreras-Vidal et al. (2018)	EEG	BrainAmpDC, Brain Products			64	1,000		Whole scalp	H2 Lower Limb Exoskeleton, EEGLAB toolbox
Tobar et al. (2018)	EEG	Active Two System, BIOSEMI	Verio fMRI Scanner (Siemens AG), EMG BrainAmp (Brain Products)		32	256	10-20		ActiView software for EEG signals, SPM8, EEGlab, Matlab
Liu et al. (2018)	EEG		3 EOG, and 4 EMGElectrodes		32	2,048	10-20	Fp1-FP2, AF3-AF4, F7-F8, F3-F4, FC1, FC5-FC6, T7-T8, C3-C4, CP1-CP2, CP5-CP6, P7-P8, P3-P4, Pz, PO3-PO4, O1,Oz, O2, FC2, Fz, and Cz	LegoPress to emulate gait movement, ActiveTwo measurement system, BioSemi
Hedian et al. (2018)	fNIRS	FOIRE-3000, Shimadzu Corp.			8 D & E	7.792	10-20	PMC, SMA, and PFC	
Khan R. A. et al. (2018)	fNIRS	DYNOT, Nix Medical Technologies			9	1.81	10-20	M1 in the left hemisphere	MATLAB
Costa-Garciacutea et al. (2019)	EEG			actiCHamp, Brain Products	31	500	10-10	Fz, FC1-FC6, FCz, C1-C6, Cz, CP1-CP6, CPz, P1-P4, Pz, PO7-PO8, PO3-PO4	
Elvira et al. (2019)	EEG		Tech MCS V3 with 7 IMUs, Technaid	USPamp, g.Tec company	32	1,200	10-10	Fz, FC1-FC6, FCz, C1-C6, Cz, CP1-Cp6, P1-P4, Pz PO7-PO8, PO3-PO4, CPZ, and Poz	MATLAB with API by gUSBamp
Li et al. (2020a)	fNIRS	LightNIRS, Shimadzu Corp.			8 E & D	13.33	10-20	PFC, PMC, and SMA	Anaconda 3.5
Li et al. (2020b)	fNIRS	LightNIRS, Shimadzu Corp.			8 D & E	13.33	10-20	PFC, PMC, SMA	LightNIRS

#### 3.1.1. EEG Signals

**Figure 3:**
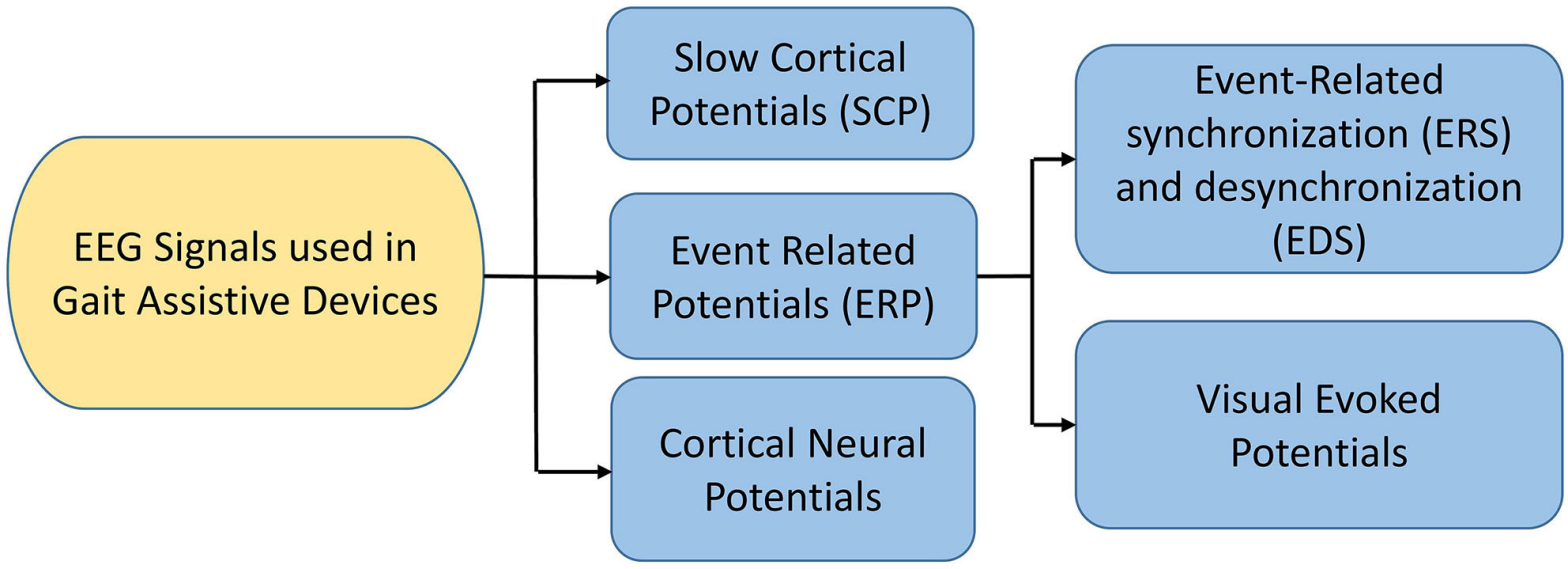
EEG signals used in BCI (Ramadan and Vasilakos, 2017; Tariq et al., 2018).

EEG is a neuro-imaging technology known for its high temporal resolution and a widely used modality for gait investigation (Lazarou et al., 2018). EEG signals record the electrical activity due to neuronal activation over a short period using multiple electrodes. Mainly two different electrodes are used to record the neuronal activity: active and passive electrode (Mathewson et al., 2017). Passive electrodes need an external amplifier to amplify small electrical activity while the active electrode has a built-in embedded amplifier (Mathewson et al., 2017). Physiological signal specially EEG have inherent challenges due to presence of significant electrical noise at low frequencies. Amplification is done to permit the signal for further processing. EEG signals are classified into six different types based on the frequency and cortical activation, as shown in Table 6. Different EEG-based BCI systems use various EEG signals, such as event-related cortical potentials, slow cortical potentials, and cortical neuronal potentials, as shown in Figure 3. The mu (μ) and beta (β), also known as sensorimotor rhythms (SMR), which are evoked event-related synchronization and de-synchronization (ERS/ERD), and are direct relation with proportional power decrease in MI or ME of lower limb movements (Kalcher and Pfurtscheller, 1995). Slow cortical potentials (SCPs) are signals with frequency low below 1 Hz. SCPs are the result of intra-cortical inputs to different cortical layers, and they are detected in frontal and central parts of the cortex (Ramadan and Vasilakos, 2017). Currently, SCPs are not a preferred alternative by many researchers due to the long training of subjects and slow variation.

**Table 6:** Types of EEG signals (Ramadan and Vasilakos, 2017).

**Type**	**Freq. (Hz)**	**Cortical activity**	**Brain area**
Delta (δ)	1-3	Found usually in babies or during the deep level of sleep and relaxation	Frontally in adults, and posteriorly in children
Theta (θ)	4-7	Unconscious, drowsiness, and idling	Frontal mid-line from Fz to Cz
Alpha (α) or Mu (μ)	7-13	Also known as (μ) when signals acquired from sensorimotor areas, Concentration and relaxation, and suppression indicates	On both sides of posterior regions with higher amplitude on the dominant side, Sensorimotor cortex
Beta (β)	12-30	Alertness, active concentration and thinking	Symmetrical distribution over sensorimotor cortex between C3 and C4
Gamma (γ)	>30	Appears for a short duration during short term memory matching objects, sounds, or tactile sensations	Somatosensory Cortex

#### 3.1.2. fNIRS Signals

**Figure 4:**
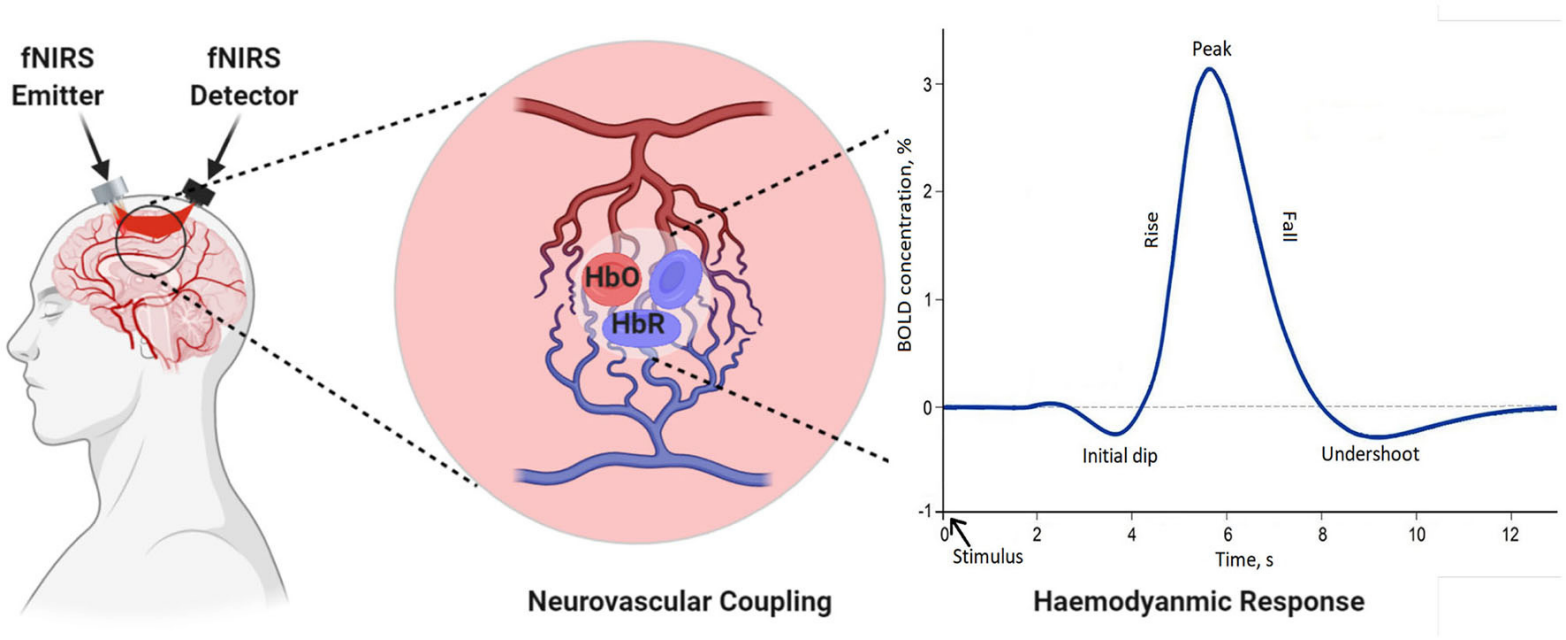
Demonstration of neurovascular coupling.

In recent years, research suggests that cerebral oxygenation and hemodynamics are affected by physical activity. To better understand the relationship between hemodynamics, physical functioning, and specific sensing, state-of-the-art neuro-imaging tools are essential. fNIRS technology is a non-invasive and powerful tool for recording hemodynamics. It helps to record the changes in oxygenated hemoglobin (HbO) and deoxygenated hemoglobin (HbR) during gait (Herold et al., 2018). The core of the theory behind measurement is based on the theory of neurovascular coupling and optical spectroscopy, as shown in Figure 4 (Leff et al., 2011; Liao et al., 2013). An increase in neural activity increases oxygen consumption to fulfill the neuronal tissue demands. When the oxygen is consumed, the process results into a decrease in HbO and an increase in HbR (Liao et al., 2013; Scholkmann et al., 2014). The reflected light that is a combination of scattered and absorbed light in the tissue, can be measured by a detector placed on the skull's surface. The change in the concentration of HbO and HbR due to neuronal activity can be calculated using the Modified Beer-Lambert Law (MBLL) 1a to 1c (Baker et al., 2014). Instrumentation of the fNIRS spectroscopy are usually based on frequency domain (FD), time-domain (TD), and continuous wave (CW) (Izzetoglu et al., 2005). In FD, tissues optical characteristics are measured by modulating the amplitude (order of tens to hundreds of megahertz) of incident light (Boas et al., 2002). TD modalities are also known as time-resolved spectroscopy (TRS). They measure the temporal information of absorption and scattering of photon distribution by introducing a pulse of light for extremely short intervals. In CW-fNIRS, absolute changes of the attenuation coefficient are determined, i.e., the difference between the emission light intensity and the detector light intensity is calculated. The obtained output fNIRS signals is a relative concentration change. The variations in optical densities are converted by MBLL into relative concentration changes of HbO and HbR. To determine relative change of concentration in HbO and HbR with the assumption of constant scattering, optical densities at two different wavelengths can be obtained by solving a set of equations according to MBLL as shown in Equations (1d) and (1e) (Kamran et al., 2015). The continuous measurements of HbO and HbR allow us to measure other markers of cortical activation, such as total hemoglobin concentration (TOI) and cortical hemodynamics (blood volume) (Obrig and Villringer, 2003). The review is focused on the description of the continuous-wave (CW) fNIRS device, which is mostly commercially used for gait investigation.

(1a) $$\begin{array}{l} A=-\log_{10}\left(\frac{I_{in}}{I_{out}}\right) \end{array}$$

(1b) $$\begin{array}{l} \Delta A\left(\lambda \right)=\varepsilon \left(\lambda \right)\times \Delta C\times d\times DPF\left(\lambda \right)+g\left(\lambda \right) \end{array}$$

(1c) $$\begin{array}{l} \Delta C=\frac{\Delta A\left(\lambda \right)}{\varepsilon \times d\times DPF\left(\lambda \right)} \end{array}$$

(1d) $$\begin{array}{l} \Delta HbO^{i}\left(k\right)=\frac{\left(\varepsilon_{HbR}^{\lambda_{1}}\frac{\Delta OD^{\lambda_{2}}\left(k\right)}{DPF^{\lambda_{2}}}\right)-\left(\varepsilon_{HbR}^{\lambda_{2}}\frac{\Delta OD^{\lambda_{1}}\left(k\right)}{DPF^{\lambda_{1}}}\right)}{l^{i}\left(\varepsilon_{HbR}^{\lambda_{1}}\varepsilon_{HbO}^{\lambda_{2}}-\varepsilon_{HbR}^{\lambda_{2}}\varepsilon_{HbO}^{\lambda_{1}}\right)} \end{array}$$

(1e) $$\begin{array}{l} \Delta HbR^{i}\left(k\right)=\frac{\left(\varepsilon_{HbO}^{\lambda_{2}}\frac{\Delta OD^{\lambda_{1}}\left(k\right)}{DPF^{\lambda_{1}}}\right)-\left(\varepsilon_{HbO}^{\lambda_{1}}\frac{\Delta OD^{\lambda_{2}}\left(k\right)}{DPF^{\lambda_{2}}}\right)}{l^{i}\left(\varepsilon_{HbR}^{\lambda_{1}}\varepsilon_{HbO}^{\lambda_{2}}-\varepsilon_{HbR}^{\lambda_{2}}\varepsilon_{HbO}^{\lambda_{1}}\right)} \end{array}$$

where *A*: signal attenuation, *I*_*in*_ and *I*_*out*_: emitted and detected light intensities respectively, Δ*A*(λ): change in signal attenuation of wavelength λ, ε(λ): extinction coefficient of a particular wavelength, Δ*C*: change in concentration of chromophore, *d*: separation between source and detector, *DPF*(λ): differential path length factor, *g*(λ): scattering at wavelength λ, Δ*HbO*^*i*^ and Δ*HbR*^*i*^: concentration changes of HbO and HbR, *i*: *i*th channel pair representation of emitter detector, λ1 and λ2: two working wavelengths of fNIRS system, Δ*OD*: variation in optical density at *k*th sample, $\varepsilon_{HbR}^{\lambda_{1}}$, $\varepsilon_{HbO}^{\lambda_{2}}$, $\varepsilon_{HbR}^{\lambda_{2}}$ and $\varepsilon_{HbO}^{\lambda_{1}}$: extinction coefficients of HbO and HbR at two different wavelengths.

#### 3.1.3. Fusion of EEG and fNIRS

Research on hybrid EEG-fNIRS is very scarce, especially for lower limb disorders. In this study, a single article was found combining EEG-fNIRS with the primary objective of testing the feasibility of using EEG-fNIRS for gait (Blokland et al., 2013). It was found that combined EEG-fNIRS modalities might be beneficial, especially for users who lack sufficient control of current EEG-based brain switches. Secondly, the MI and ME task performance was evaluated for tetraplegia patients with an accuracy of 79 and 87%. The highest classification accuracy was found by using EEG with HbR. Berger et al. (2019) found that fused EEG-fNIRS gives detailed information, both spatial and temporal, correlated with brain activity. Li et al. (2020c) developed an EEG-informed fNIRS analysis framework to improve fNIRS GLM estimation performance and investigated how different EEG rhythmic modulations are independently related to changes in the hemodynamic response during a motor execution task. The analysis showed an understanding of the inherent correlation between neural activity and hemodynamic response. Fused EEG-fNIRS can characterize complex neurovascular coupling mechanisms associated with gait disorders; it helps measure neuroplastic changes due to robot-assisted gait rehabilitation. Fazli et al. (2012), for the first time, used EEG-fNIRS in BCI and found that it increases classification accuracy. The existing literature on hBCI of EEG-fNIRS includes task classification (usually using LDA, SVM as a classifier) related to imaginary motor (Buccino et al., 2016). ME tasks are related to the upper limb, which confirms that it helps enhance classification accuracy as well as the number of control commands (Khan et al., 2014). The only disadvantage of using EEG with fNIRS is the delayed response of fNIRS compared to EEG (Zama et al., 2019). However, the disadvantage can be overcome using the detection of the initial dip instead of hydrodynamic response (Hong and Zafar, 2018).

#### 3.1.4. Fusion EEG and fNIRS With Other Bio-Signals

In hBCI different signals are recorded to help artifact removal, better classification, or enhance the number of command generation. Eye blinks influence the brain signals, and hence, in several studies, electrooculography (EOG) recordings are used to remove ocular movement artifacts from EEG data (Mingai et al., 2015). The results show that the removal of ocular artifacts improves performance in comparison to using individual modality (Fatourechi et al., 2007). Liu et al. (2018) used three EOG electrodes below outer canthi and above nasion to record the eye movement while using a regression-based approach to remove EOG artifacts. Similarly, in another study, EOG signals were recorded from two pairs of bipolar electrodes to detect vertical and horizontal eye movements, and then artifacts were removed using a regression algorithm (Li et al., 2016). In this perspective, artifacts removal EOG also plays a role in controlling the BCI system, especially for locked-in syndrome (LIS) patients. The fusion of EMG signals with EEG signals is user-specific and depends upon the task performed.

In most of the applications, EEG with EMG was used to control assistive device and intention decoding. Bulea et al. (2014) synchronized EEG with EMG for decoding sitting and standing intentions. EMG sensors were placed bilaterally on the tibia anterior, biceps femoris, gastrocnemius, and vastus lateralis to record movement onset of each sit to stand and stand to sit transition. A combination of EMG signals helped to get better classification accuracy and can be used for rehabilitation. Bulea et al. (2014) used the EMG with EEG to increase the classification accuracy by recording EMG data from lower extremity muscular movements and the onset of each stand-to-sit and sit-to-stand transition. Tobar et al. (2018) used the EMG electrode to ensure task execution during decoding of ankle flexion and extension.

**Figure 5:**
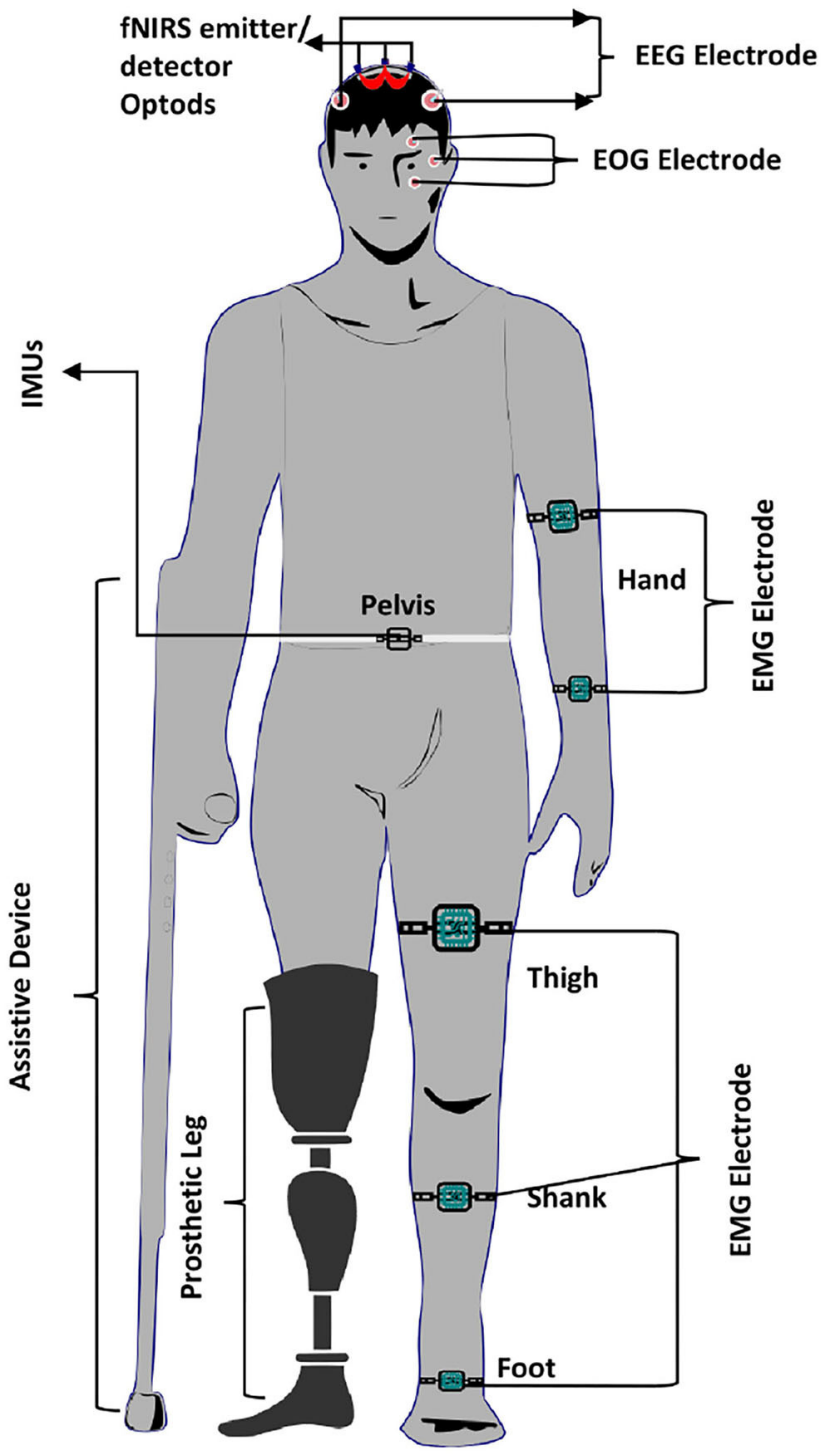
Illustration of bio-sensors placement during gait assessment.

Similarly, Liu et al. (2018) decode lower limb movement using a pair of EMG electrode on tibialis posterior muscle and EOG signals recording and motor intention by EEG. Synchronization of all these signals in continuous and online performance makes it feasible for practical brain switch closed-loop BCI. Gui et al. (2017) used EMG to enhance active participation in rehabilitation with cognitive and physical BCI setup. EMG signals also play an essential role in serving as a torque predictor to provide information about torque for controller (Rosen et al., 2001; Gui et al., 2019). For smooth operation of BCI, a pair of EMG electrodes are mounted on tibialis anterior muscles to monitor that the subject did not contract the legs during BMI operation (Liu D. et al., 2017). Athanasiou et al. (2017) used EMG with bipolar EEG Ag/AgCl electrodes on the top left and right tibialis anterior muscle to detect the intention to walk. A combination of EEG and EMG signals can be utilized to define the control strategy of the robotic assistive system (Villa-Parra et al., 2015). Inertial sensors are widely used as wearable sensors for analysis of gait and balance problems. This type of sensor is validated for both groups of healthy and motor impairment (Mason et al., 2014). Inertial sensors are used to measure: acceleration, velocity, gravitational force, and orientation. Hortal et al. (2016) used seven inertial measurement units (IMUs) to obtain different subject movement parameters. The system is used to detect gait changes to train better and evaluate the accuracy (Salazar-Varas et al., 2015). In many online BCI systems, IMUs are used to measure the real movement to ensure smooth operation. Elvira et al. (2019) placed 7 IMUs on lumber, thigh, shin, and foot to measure the actual movement to detect stop intention. IMUs serve as feedback to ensure the real physical stop. Zimmermann et al. (2013) used fNIRS with blood pressure sensor, respiration sensor, skin conductance response (SCR), and electrocardiogram (ECG) for detection of ME. It was found that hybridization helped to increase the classification accuracy from 79.4 to 88.5%. Tobar et al. (2018) used fMRI with EEG and EMG to identify the exact location of brain area activation. Figure 5 proposed possible brain imaging and sensor placement to better understand human kinematics and dynamics for future works.

### 3.2. Signal Pre-processing

Signal pre-processing is an important step to remove experimental, instrumental, and physiological noise. It is also an important step to pre-process the signal to get the signal's best discriminating feature. In investigating gait disorder, one of the common types of noise is due to motion. Some conventional filters used to deal with such noise and other techniques to pre-process the signal are discussed as follows.

#### 3.2.1. Common Average Reference (CAR)

##### 3.2.1.1. Background

CAR is a conventional filter used to smooth the basal brain activity contribution and useful for real-time applications (Ludwig et al., 2009; Bulea et al., 2015; Beurskens et al., 2016). The basic idea for re-referencing the signal to CAR is performed by subtracting the average value of samples of all electrodes to each sample. It can be computed as Equation (2):

(2) $$\begin{array}{l} y_{i}\left(t\right)=x_{i}\left(t\right)-\frac{1}{M}\overset{M}{\underset{m=1}{\sum }}x_{m}\left(t\right) \end{array}$$

where *y*_*i*_(*t*): output filtered signals, *x*_*m*_(*t*): the recorded sample at instant *t* of each electrode, *T*: total number of data points, *M*: total number of the electrode. Another common spatial filter used after the CAR filter is a weighted average filter (WAVG). It can be applied as a small Laplacian calculated using Equation (3).

(3) $$\begin{array}{l} e_{i}\left(t\right)=e_{i}\left(t\right)+\frac{1}{K}\overset{K}{\underset{j=1}{\sum }}e_{j}\left(t\right) \end{array}$$

where *e*_*i*_(*t*) is the *i*th channel, and *K* is the number of closest neighbor channels.

##### 3.2.1.2. Application

WAVG works well in improving the performance of EEG slow cortical potential detection. It is commonly used in EEG studies to remove the global background activities (Salazar-Varas et al., 2015; Liu D. et al., 2017; Liu et al., 2018). Other studies are using CSP to extract spatial patterns that help classify hybrid EEG-fNIRS signals (Fazli et al., 2012; Buccino et al., 2016; Ge et al., 2017; Kwon et al., 2020).

#### 3.2.2. Artifact Subspace Reconstruction (ASR)

##### 3.2.2.1. Background

ASR utilizes a sliding window technique. Each window of EEG data is divided using principal component analysis (PCA) to be compared statistically with data from noise-free baseline EEG recordings. For each sliding window, ASR algorithms recognize principal subspace, which deviates from the noise-free baseline EEG data (Bulea et al., 2014).

##### 3.2.2.2. Application

The ASR algorithm is usually sufficient for removing the physiological noise of large amplitude, such as large amplitude movements, ocular artifacts, and typical muscle burst in EEG signals (Zhang et al., 2017; Contreras-Vidal et al., 2018; Tortora et al., 2020). Bulea et al. (2015) used ASR to remove high amplitude artifact from the EEG recorded for speed control during walking.

#### 3.2.3. Independent Component Analysis

Independent component analysis (ICA) is a statistical and computational technique that can remove physiological noise from raw signals allowing the restoration of the original signal. ICA assumes that the input signals are mixtures of different independent components (ICs) signals generated from different cognitive activities or artifacts (Hyvärinen and Oja, 2000). Their spectral densities identify ICs associated with noise signals. ICA is a gold-standard technique to attenuate motion artifacts with various extended versions like independent vector analysis and independent low-rank matrix analysis (Kanoga et al., 2020).

#### 3.2.4. Mathematical Morphology Filter (MMF)

MMF was primarily used for ECG signals. It performs well in terms of filtering characteristics, low computational burden, low signal distortion ratio, good noise suppression ratio, and baseline correction ratio (Sun et al., 2002). However, recently it has been used in a NIRS study to remove zero drift in cerebral hemoglobin (Hedian et al., 2018). Corrosion and expansion are the two primary operations in this method. MMF can be a mathematical computed using Equation (4).

(4) $$\begin{array}{l} y=x-\frac{filter_{oc}\left(x\right)+filter_{co}\left(x\right)}{2} \end{array}$$

where *filter*_*oc*_(*x*) and *filter*_*co*_(*x*) are known as open-close and close-open filters and can be calculated from corrosion and expansion operations, respectively (Hedian et al., 2018; Li et al., 2020a).

#### 3.2.5. Wavelet-Minimum Description Length (Wavelet-MDL)

Jang et al. (2009) proposed a wavelet-MDL, which can be used to overcome the problem of noise due to cardiac, breathing, vasomotion, and other experimental noises in fNIRS data. In this method, wavelet transformation is applied to time series data obtained from NIRS to decompose it into bias, hemodynamic signal, and noise signal in distinct scales. Experimental results show that wavelet-MDL performed well-compared to the conventional approaches (Rea et al., 2014). The wavelet-MDL de-trending algorithm can remove possible global trends in fNIRS signals due to the heartbeat, breathing, or vasoconstriction (Beurskens et al., 2014).

#### 3.2.6. SHADE

Ambient light can significantly affect fNIRS' signal quality, mainly if the experiment is not conducted in a controlled environment. Sherkat et al. (2020) recently introduced an efficient empirical compensation algorithm to mitigate the non-stationary impact of ambient light on the fNIRS data. In this approach, the system dynamically measures the ambient light. Then, in the post-processing part, the measured signal was used to eliminate ambient light's effect using a sequential change points detection algorithm (Hawkins and Deng, 2010) and to construct a trend line. SHADE can help to apply fNIRS beyond the lab environment.

#### 3.2.7. Canonical Correlation Analysis (CCA)

CCA is a statistical method and multivariate form of the generalized linear model. It is used to investigate the relationship among two or more sets of variables, each set consisting of at least more than one variable (Thompson, 2005). Al-Shargie et al. (2017) applied CCA to the fused EEG-fNIRS signal at the feature level by maximizing the inter-subject covariance across modalities. Multiple CCA variants are introduced in neuroscience because of its robust characterization of jointly investigating the relationship between various data sets.

#### 3.2.8. Algorithms for Selecting Region of Interest (ROI)

It is evident if the brain activity's exact location is known, it will reduce the computational and hardware complexity (use of multiple electrodes/optodes) (Hong et al., 2018). The most common approach adopted in EEG/fNIRS/hybrid EEG-fNIRS-based BCI studies is channel averaging. In this technique, we average all the channels used for detecting brain activity. The method is beneficial if brain activation appears in most of the channels. But if brain activity appears in very few channels, in that case, the magnitudes of the peaks reduce due to an inactive channel, which ultimately reduces the performance. The technique is not suitable for data acquired from locked-in syndrome patients (Hong et al., 2018). There is another approach, known as averaging over a local region, which better than universal averaging. In this technique brain region is divided into sub-regions (Abibullaev and An, 2012; Aghajani et al., 2017). In case when the stimulation paradigm is known, then a t-values-based channel can be a suitable method for channel selection. A t-value-based channel selection method means that only those channels will be selected for further processing with positive t-values. Another way of doing this can be by using the baseline correction. Instead of computing the t-values for individual channels, maximum values for rest and task period are compared, channels with positive t-value are selected.

#### 3.2.9. Other Common Filters

**Figure 6:**
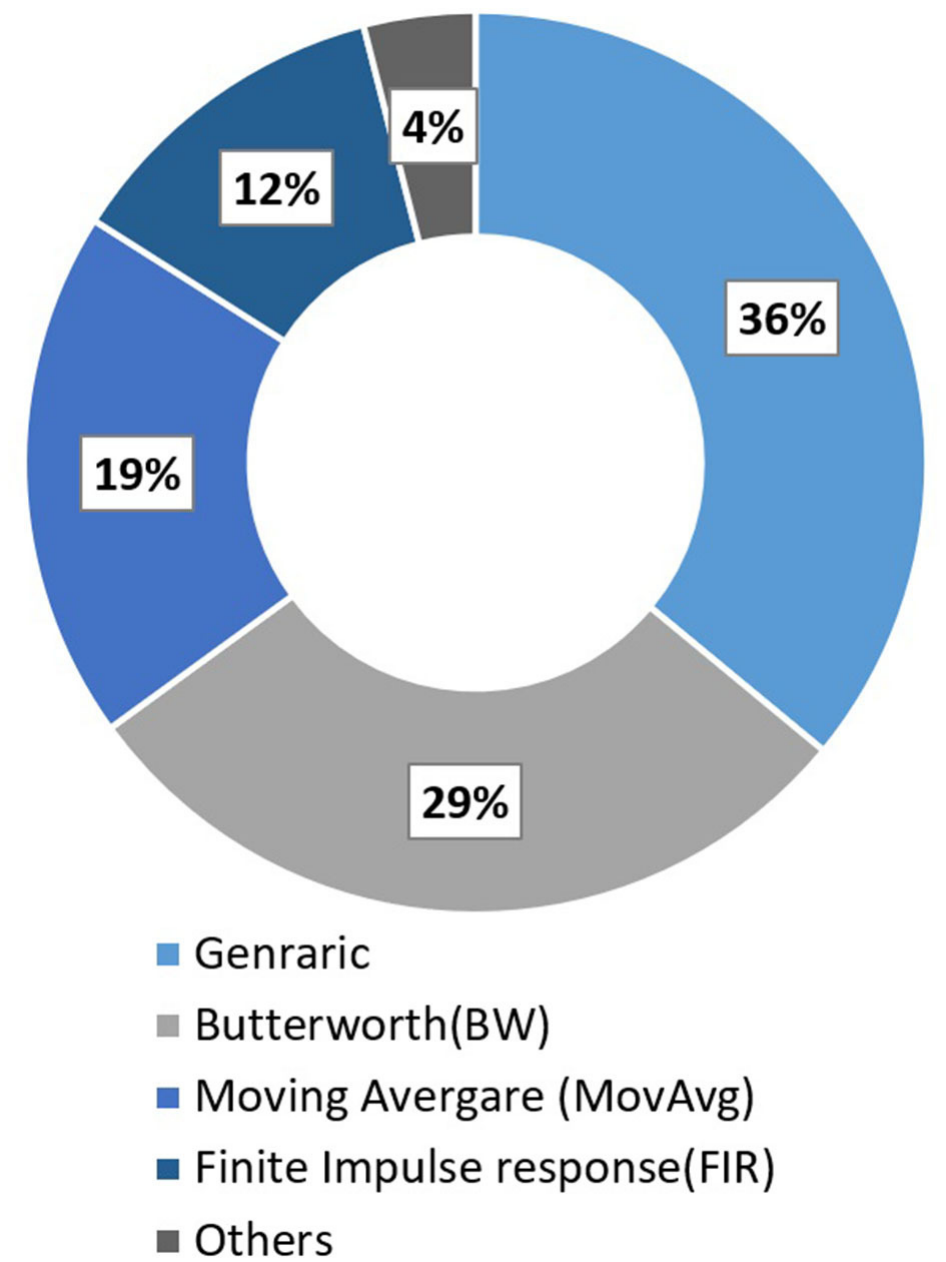
Filter used in fNIRS studies in 2016 (Pinti et al., 2019).

Pinti et al. (2019) reviewed 110 different fNIRS studies to investigate the current status and issues regarding the pre-processing of fNIRS data. Some conventional deployed filters along with their respective percentage usage in all these fNIRS studies are Butter-worth (BW) (28.8%), moving average (18.8%), finite impulse response (12.5%), and wavelet minimum description length (3.8%). Furthermore, a complete description can be found in Figure 6. Most of the filters have the characteristic of either band-pass filter (61.3%) or low pass filter (59.7%). Some other filter commonly used are Z-score based artifacts removal (Lopez-Larraz et al., 2016; Li et al., 2020b), regression analysis algorithm (Li et al., 2016), BW (Bulea et al., 2014; Hortal et al., 2016; Gui et al., 2017; Zhang et al., 2017; Contreras-Vidal et al., 2018; Liu et al., 2018), Chebyshev filter (Li et al., 2020a,b), Kalman filters (Khan R. A. et al., 2018). However, some recent studies showed high inter-subject variability and recorded the difficulty in eliminating gait-related movement artifacts from EEG signals (Kline et al., 2015; Snyder et al., 2015; Bradford et al., 2016; Nathan and Contreras-Vidal, 2016). Croce et al. (2017) introduced a new filtration method known as Particle filter based on Bayesian sequential Monte Carlo. They show feasibility and improvements to combining EEG-fNIRS recordings. Therefore, future research should focus on improving the methods and techniques to remove these artifacts.

### 3.3. Features Extraction and Selection Methods

Feature extraction and selection methods have core importance in BCI systems as they hugely impact the classifier's performance, which ultimately generates control commands. Some core techniques of feature extraction and selection used in EEG and fNIRS-based studies are discussed in this section.

#### 3.3.1. Principle Component Analysis (PCA)

##### 3.3.1.1. Background

PCA is commonly used for dimensionality reduction and statistical feature extraction. PCA resorts to a linear transformation to convert input data (possibly correlated) to uncorrelated variable data set called principal components (Wold et al., 1987). Principal components generated by linear transformation are sorted so that the first principal component has the highest possible variance. This variance allows input brain signal to be separated into different components (Nicolas-Alonso and Gomez-Gil, 2012). The computation equation for PCA is shown in Equations (5a) to (5c).

(5a) $$\begin{array}{l} \sum =\overset{n}{\underset{i=1}{\sum }}\left(P_{i}-m\right){\left(P_{i}-m\right)}^{t} \end{array}$$

(5b) $$\begin{array}{l} m=\frac{1}{n}\overset{n}{\underset{i=1}{\sum }}P_{i} \end{array}$$

(5c) $$\begin{array}{l} V=A^{t}\left(q-m\right) \end{array}$$

where ∑ is the covariance matrix, *n* is the number of samples, *m* is mean vector, *P*_*i*_ is training sample, *V* feature vector, and *q* is the test data. PCA computes *V* from the data *A* by projecting test data *q* onto a new subspace.

##### 3.3.1.2. Application

Contreras-Vidal et al. (2018) applied PCA to reduce the dimensionality of large data set keeping variance (about 99%) in the original data set retained; hence it significantly helps to reduce the computational complexity. Similarly, Li et al. (2020a) used PCA to reduce the dimensionality of data acquired for self-regulated intention detection using fNIRS, keeping 95% of the selected feature space variance. Do et al. (2013) used class-wise PCA and approximate information discriminant analysis for dimensionality reduction of EEG data for gait orthosis.

#### 3.3.2. Autoregression Model (AR)

##### 3.3.2.1. Background

In an AR model, we predict variables of interest using previous values of the variable. AR models the signals as the random output signal of linear time-invariant filter, where input is noise is modeled with mean and variance of zero and σ^2^. The goal of the AR approach is to obtain filter coefficients because it is assumed that different thinking activities produce different filter coefficients. Filter modules are used as a feature of the signal. Mathematically the output signal *y*_*t*_ of the AR model can be written using Equation (6).

(6) $$\begin{array}{l} y_{t}=c+\phi_{1}{\;y}_{t-1}+\phi_{2}{\;y}_{t-2}+.........+\phi_{p}{\;y}_{t-p}+\varepsilon_{t} \end{array}$$

where *p* is the order of model, ϕ_*i*_ is the *i*th filter coefficients, ε_*t*_ is the noise. The resulting filter coefficients can be used to estimate the power spectrum of EEG signal using Equation (7).

(7) $$\begin{array}{l} y\left(\omega \right)=\frac{1}{|\sum_{k=1}^{p}\phi_{k}e^{-jk\omega }{|}^{2}} \end{array}$$

where ϕ_*k*_ is estimated filter coefficients.

##### 3.3.2.2. Application

Lopez-Larraz et al. (2016) applied a 16th-order AR model to EEG data for feature extraction. Due to the continuous nature of EEG signals, the multivariate adaptive AR (MVAAR) model has been used to extract features from EEG signals for online BCI systems more efficient (Anderson et al., 1998). Wang et al. (2010) applied MVAAR for the classification of MI, showing that MVAAR is a valuable adaptive method for feature extraction. Costa-Garciacutea et al. (2019) used auto-regressive spectral analysis based on the method of maximum entropy. AR-parameters were calculated by reducing the sum of square forward and backward estimation errors.

#### 3.3.3. Common Spatial Pattern (CSP)

##### 3.3.3.1. Background

CSP is a common feature extraction method applied to EEG signals. CSP designs spatial filters for time series data in such way that the variances in the data are optimal for discrimination (Rao and Scherer, 2010). CSP aims to make the classification more efficient by applying the spatial filter, which transforms the input signal to output signal with optimal variance for better classification (Ramoser et al., 2000). Spatial covariance matrix *C*_*var*_ is calculated from input raw single matrix *x*_*t*_ of the *N* × *T* dimension using Equation (8) (Nicolas-Alonso and Gomez-Gil, 2012)

(8) $$\begin{array}{l} C_{var}=\frac{x_{t}x_{t}^{\prime }}{Tr\left(x_{t}x_{t}^{\prime }\right)} \end{array}$$

where *Tr* is the trace of $x_{t}x_{t}^{\prime }$ matrix, *N* is the number of samples per channel, and *T* is the number of channels. For *i* = 1, 2, ..*n* class problem, CSP calculates spatial covariances matrix for both the class and compute composite spatial covariance matrix *C*_*c*_ by adding spatial co-variance of both classes $C_{c}=\bar{C_{1}}+\bar{C_{2}}$. *C*_*c*_ matrix is real and symmetric; it is factorized to $C_{c}=U_{c}\lambda_{c}U_{c}^{\prime }$, where *U*_*c*_ is a matrix of eigenvectors, and λ_*c*_ is the diagonal matrix of eigenvalues. Applying whitening transform in Equation (9).

(9) $$P=\sqrt{\lambda_{c}^{-1}}U_{c}^{\prime }$$

All eigenvalues of $P{\bar{C}}_{c}P\prime$ are equal to unity, where $\bar{C_{1}}$ and $\bar{C_{2}}$ are transformed using $S_{1}=P{\bar{C}}_{1}P\prime$ and $S_{2}=P{\bar{C}}_{2}P\prime$, respectively. *S* represents the shared matrix for each class. For each class, the eigenvectors having the largest eigenvalues for one class correspond to the smallest eigenvalue of other class and vice versa. Finally, the feature vector for input signal *x*_*t*_ is computed as *Z* = *WE* where *W* = (*B*′*P*)′, a spatial filter matrix built by CSP procedure.

##### 3.3.3.2. Application

The selection of time window significantly affects the CSP's performance, which is either selected experimentally or manually. However, Jiang et al. (2020) proposed an optimized way of feature selection from temporal pattern combinations, which can solve the problem of time window selection. CSP enhances the accuracy of synchronous BCI, where the signal is only transmitted at predefined intervals. However, the CSP does not provide the same results for asynchronous BCIs. It can be explained due to the non-linear properties of EEG signals (Mousavi et al., 2011). Salazar-Varas et al. (2015) used CSP to extract features from EEG signals to detect unexpected obstacles during walking. Several other improved versions of CSP were proposed in the literature to enhance the performance such as wavelet common spatial pattern (WCSP) (Mousavi et al., 2011), common spatiospectral pattern (CSSP) (Lemm et al., 2005), and common sparse spectral spatial pattern (CSSSP) (Dornhege et al., 2006).

#### 3.3.4. Wavelet Transform (WT)

##### 3.3.4.1. Background

WT is a mathematical method for extracting information from time-frequency domain signals. Wavelets are functions of different frequencies and finite duration, allowing the signal's simultaneous study in both time and frequency domain contrarily to other signal analysis methods such as the Fourier transform (Samar et al., 1999). The Fourier transform only provides an analysis of the signal activity in the frequency domain. Using a modulated window and the signal at different scales, the WT overcomes the drawback of Fourier transform by decomposing the signal in both the time and frequency domain at multiple scales. The essential concepts behind the wavelet transform are scaling and shifting. The two significant transforms in wavelet analysis are continuous wavelet transform (CWT) and discrete wavelet transform (DWT). CWT is defined as the signal convolution *x*(*t*) with wavelet function Ψ_(*s*,τ)_(*t*) (Samar et al., 1999). It can be computed from Equation (10).

(10) $$\begin{array}{l} w\left(s,\tau \right)=\int_{-\infty }^{+\infty }x\left(t\right){\text{Ψ}}_{\left(s,\tau \right)}^{*}\left(t\right)dt \end{array}$$

where *w*(*s*, τ) is wavelet coefficient in which *s* is scale and τ is the time of wavelet function ${\text{Ψ}}_{\left(s,\tau \right)}^{*}\left(t\right)$, while * indicates complex conjugation. Ψ_(*s*,τ)_(*t*) in Equation (11) is wavelet function, which is dilated and shifted form of mother wavelet Ψ(*t*). The mother wave must satisfy the condition of Equation (12).

(11) $$\begin{array}{l} {\text{Ψ}}_{\left(s,\tau \right)}\left(t\right)=\frac{1}{\sqrt{s}}\text{Ψ}\left(\frac{t-\tau }{s}\right) \end{array}$$

(12) $$\begin{array}{l} \int_{-\infty }^{+\infty }\text{Ψ}\left(t\right)dt=0 \end{array}$$

##### 3.3.4.2. Application

CWT introduces a lot of complexity and redundancy because it incorporates signal analysis with the highest number of frequencies using multiple dilations and mother wavelet transforms. DWT reduces this complexity and redundancy and dilates and translates the mother wavelet into specific discrete values only (Burke-Hubbard, 1998). The use of WT requires the selection of the mother wavelet. Different mother wavelets can be found in BCI, and the selection of any one of them depends upon the type of data that needs to be removed from the signal. CWT can be used to extract important brain hemodynamics features efficiently at multiple frequencies subjected to the appropriate selection of mother wavelet function (Abibullaev and An, 2012). WT is also widely used to remove ocular artifacts and feature extraction form EEG data (Krishnaveni et al., 2006; Kumar et al., 2008; Khushaba et al., 2010; Hsu et al., 2012; Chen et al., 2015).

#### 3.3.5. Genetic Algorithm (GA)

##### 3.3.5.1. Background

**Figure 7:**
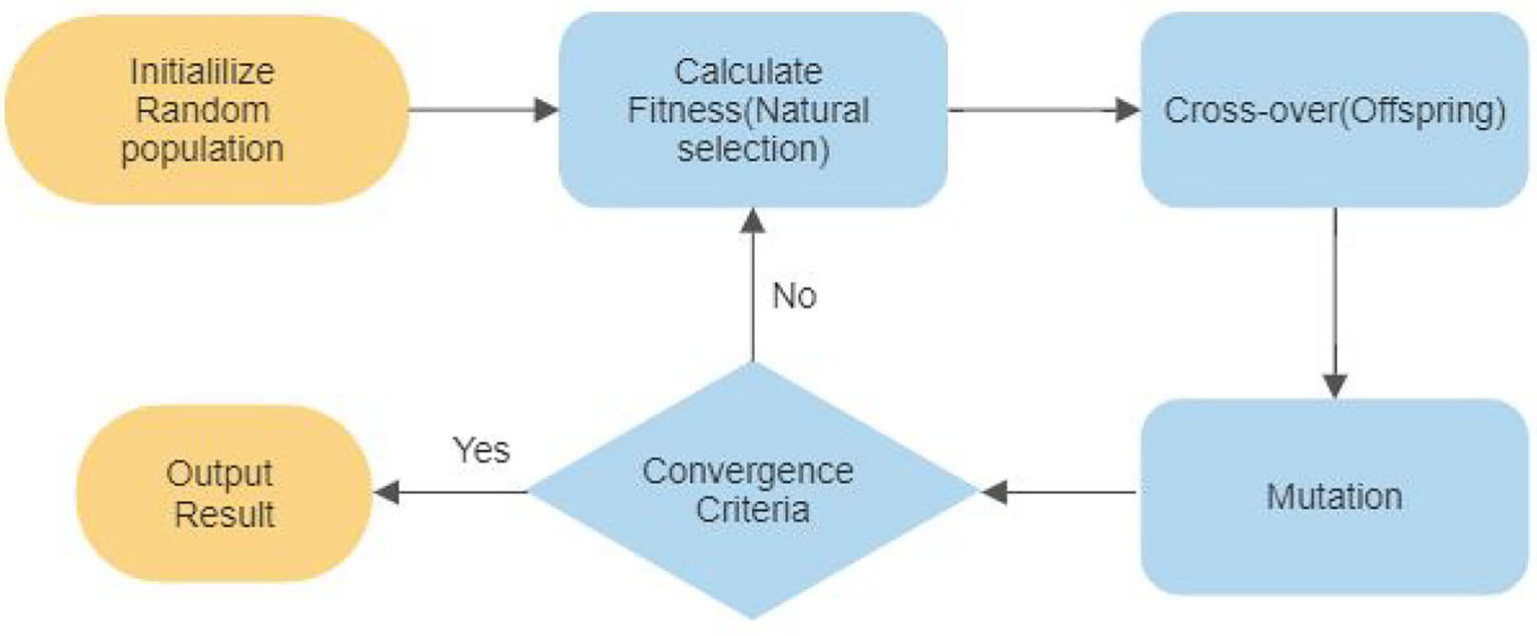
Genetic algorithm process flowchart.

GA is an optimization technique, which is widely used for auto-selection of optimal features. The algorithm's core is the candidate solution population from the initial population and then coded into a binary string known as a chromosome. The initial population is usually randomly generated in case previous possible solutions are not available. The steps followed in GA are explained in the flow chart shown in Figure 7. Every individual chromosome is evaluated according to a fitness function. The selection of mating chromosomes is made stochastically to keep the diversity in the population. After the selection of mating poles, cross-over is performed randomly to get new offspring. The same process is repeated for every new generation until an acceptable solution is reached.

##### 3.3.5.2. Application

The hybridization of a genetic algorithm with SVM is applied for optimal feature selection from fNIRS signals to produce the best result (Noori et al., 2017). Li et al. (2020a) used both single and double layer-GA-SVM model to classify four different types of self- regulated gait intentions. The double-layer GA based SVM model showed an accuracy of 13.8% higher than the single-layer SA-SVM model.

#### 3.3.6. Sliding Window Method

The sliding window is transformed using ASR with PCA to identify high variance channels by statistical comparison with minimal movement artifact EEG data recorded using EEG for balance control (Bulea et al., 2015). Ghonchi et al. (2020) used a sliding window method to exploit temporal information of the EEG-fNIRS signals and add it to three-rank tensor (DNNs). The sliding window size affects the performance of the classification algorithm directly (Ghonchi et al., 2020).

#### 3.3.7. Features in Hybrid Modalities

Two primary BCI modalities used in mobile BCI applications are EEG and fNIRS (Hong et al., 2018). Power spectral density method used in most of the EEG-fNIRS studies for classification of features (Putze et al., 2014; Tomita et al., 2014). It uses strength of signal as function of frequency. Few other studies used the time-frequency phase, and the coefficients of a wavelet transform as features for EEG, which were combined with fNIRS for hybridization (Yin et al., 2015; Li et al., 2017). Band power and logistic regression coefficients are used as features in hybrid EEG-fNIRS study for tetraplegia patients (0–15 s and 3–18 s window for EEG and fNIRS, respectively) (Blokland et al., 2013).

#### 3.3.8. Other Common Features

In fNIRS studies *HbO*, *HbR* and *HbT* concentrations are commonly used as features in most of the fNIRS-based BCI studies (Blokland et al., 2013; Hedian et al., 2018; Li et al., 2020b). Some other common time domain feature used are: signal mean (SM), signal skewness (*SK*), kurtosis (*Z*), signal variance (*Var*), and signal peak show in Equations (13b) to (13d) (Naseer et al., 2016; Aghajani et al., 2017; Khan and Hong, 2017; Li et al., 2017, 2020a; Hong et al., 2018; Khan R. A. et al., 2018; Shin, 2020).

(13a) $$\begin{array}{l} SM=\frac{1}{N}\overset{N}{\underset{i=1}{\sum }}Z_{i} \end{array}$$

(13b) $$\begin{array}{l} SK\left(Z\right)=E\left[\begin{array}{c} {\left(\begin{array}{c} \frac{Z-\mu }{\sigma } \end{array}\right)}^{3} \end{array}\right] \end{array}$$

(13c) $$\begin{array}{l} Kurtz\left(Z\right)=E\left[\begin{array}{c} {\left(\begin{array}{c} \frac{Z-\mu }{\sigma } \end{array}\right)}^{4} \end{array}\right] \end{array}$$

(13d) $$\begin{array}{l} Var\left(Z\right)=\frac{\sum {\left(Z-\mu \right)}^{2}}{N} \end{array}$$

where, *N* is total number of observations, *Z*_*i*_ is Δ*C*_*HbO*_(*t*) across each observation, σ is standard deviation and *E* is expected value of *Z*, respectively. Usually such features are scaled between 0 *min*(*sf*) and 1 *max*(*sf*) using Equation (14).

(14) $$\begin{array}{l} sf^{\prime }=\frac{sf-min\left(sf\right)}{max\left(sf\right)-min\left(sf\right)} \end{array}$$

where *sf*′ and *sf* are scaled feature and original features. Other common applied filters are CSP, power, slop and polynomial (Salazar-Varas et al., 2015).

### 3.4. Classification Algorithms

Based on the feature extracted from the brain signals, classifiers play a vital role in discriminating various tasks. The fusion of *EEG*+*fNIRS* significantly increases the classification accuracy and enhance the number of commands (Fazli et al., 2012; Kaiser et al., 2014; Khan et al., 2014; Yin et al., 2015; Hong and Khan, 2017; Li et al., 2017; Liu Y. et al., 2017; Abtahi et al., 2020; Cicalese et al., 2020). Table 7 is evidence of enhancement in classification accuracy of using hybrid EEG-fNIRS signals for MI and ME tasks. Xie et al. (2014) reviewed the hybrid soft computing methods used for the classification of bio-signals and concluded that these methods help improve classification accuracy. Since no particular article focusing or proposing a classification algorithm for hybrid EEG-fNIRS with the application of gait was not found during the article's review, therefore, conventional and modern classifiers used in the literature specifically for the classification of gait activities (using EEG and fNIRS) are discussed in the following sections. Usually, for hybrid EEG-fNIRS studies, these conventional classifiers are commonly used.

**Table 7:** Summary of fused EEG-fNIRS studies for motor task.

**References**	**Main finding**
Fazli et al. (2012)	Concurrent measurements of EEG and fNIRS can significantly improve the BCI systems classification accuracy and performance for sensory-motor rhythm.
Buccino et al. (2016)	Classification of four different hand movements is executed. Different features were compared to diminish the fNIRS delay in change detection using common spatial patterns and genetic algorithms.
Ge et al. (2017)	The study stepped forward toward real-time BCI application by using a few EEG and fNIRS channels to improve the hybrid BCI system's classification accuracy for the imaginary motor task by improving the signal acquisition (source analysis) and signal processing (phase-space reconstruction).
Li et al. (2017)	The classification accuracy for hybrid EEG-fNIRS is enhanced by integrating their complementary properties and early temporal features.
Khan M. J. et al. (2018)	A novel classifier based on a modified vector phase diagram is proposed for the finger-tapping task. The results suggest an enhancement in classification accuracy with the proposed method using a time of 1.5 s.
Chiarelli et al. (2018)	DNNs show better classification accuracy for EEG-fNIRS recording than LDA and SVM while performing left and right-hand imagery tasks.
Kwon et al. (2020)	The study proves the feasibility of achieving higher classification with less EEG electrodes and fNIRS optodes than the bulky individual EEG and fNIRS based BCI system.

#### 3.4.1. Linear Discriminant Analysis (LDA)

##### 3.4.1.1. Background

The original linear discriminator for two problems was introduced by Ronald A. Fisher (1936) and is still an effective approach for dimensionality reduction and pattern classification. LDA reduces the feature dimensionality into a smaller subspace with good class separability while preserving the original information (Lotte et al., 2007). LDA assumes that the data comes from a normal distribution and obtains hyper-plane, which minimizes the inter-class while maximizing the distance between two class's means. LDA searches for vector *v* in feature space such that when two classes are projected, they are well-separated. An eigenvalue problem is solved to calculate a vector *v* from objective function *J*(*v*), which is governed by between class (*S*_*b*_) and within-class scatter (*S*_*w*_) matrices, as shown in Equations (15a) to (15e).

(15a) $$\begin{array}{l} J\left(v\right)=\frac{v^{t}S_{b}v}{v^{t}S_{w}v} \end{array}$$

(15b) $$\begin{array}{l} S_{b}=\left(m_{1}-m_{2}\right){\left(m_{1}-m_{2}\right)}^{T} \end{array}$$

(15c) $$\begin{array}{l} S_{w}=\underset{X_{n}\in C_{1}}{\sum }\left(X_{i}-m_{1}\right){\left(X_{i}-m_{2}\right)}^{T}+\underset{X_{n}\in C_{2}}{\sum }\left(X_{i}-m_{1}\right){\left(X_{i}-m_{2}\right)}^{T} \end{array}$$

(15d) $$\begin{array}{l} \lambda v=S_{w}^{-1}\left(m_{1}-m_{2}\right) \end{array}$$

(15e) $$\begin{array}{l} v=S_{w}^{-1}\left(m_{1}-m_{2}\right) \end{array}$$

where *X*_*n*_ denotes samples, *m*_1_, and *m*_2_ are means of respective classes 1 (*C*_1_) and 2 (*C*_2_). The largest eigenvalue in eigenvector obtained from Equation (15e) will be optimal *v*.

##### 3.4.1.2. Application

Due to LDA's simplicity and effectiveness, it is widely used in the classification of EEG and fNIRS signals for gait disorders (Bulea et al., 2014; Rea et al., 2014; Salazar-Varas et al., 2015; Naseer et al., 2016; Gui et al., 2017; Khan R. A. et al., 2018; Costa-Garciacutea et al., 2019; Elvira et al., 2019). Fazli et al. (2012) used LDA to classify MI tasks and found that simultaneous EEG-fNIRS measurement helped increase the classification accuracy by 5%. In most EEG-fNIRS studies, a multiclass problem was decomposed into a pairwise classification problem, and then binary classification is performed using LDA (Kwon et al., 2020). Other studies also showed LDA's effectiveness for fused EEG-fNIRS in various other applications (Khan and Hong, 2017; Liu Y. et al., 2017; Cicalese et al., 2020).

#### 3.4.2. Sparse Logistic Regression (SLR) and Sparse Discriminant Analysis (SDA)

##### 3.4.2.1. Background

SLR is the Bayesian extension of logistic regression. The SLR combines the logistic regression with automatic relevance determination to perform feature selection and model training for classification simultaneously. SDA is a method of performing LDA with a sparseness criterion enforced, so that feature selection and classification are performed simultaneously (Lopez-Larraz et al., 2016). SDA is based on the optimal scoring interpretation of the LDA.

##### 3.4.2.2. Application

Tobar et al. (2018) used SLR to classify ankle flexion and extension at two different force levels. It performs well in the presence of irrelevant features compared to other popular classification algorithms such as SVM. The method shows the accuracy of 65.64% for the classification of nine class EEG data.SDA can be extended to perform sparse discrimination via mixtures of Gaussians if boundaries between classes are non-linear or if subgroups are present within each class (Clemmensen et al., 2011).

#### 3.4.3. Support Vector Machine (SVM)

##### 3.4.3.1. Background

SVM is among the most commonly used classifiers in investigating gait disorders and rehabilitation. SVM tries to find an optimal hyperplane, which maximizes the distance between the nearest training points known as support vectors. The optimal value of r^*^ in the 2D hyperplane equation shown in Equation (16a), which maximizes the distance between the hyperplane, can be obtained from the objective function shown in Equations (16b), (16c) to (16e) are the constrain equations.

(16a) $$\begin{array}{l} f\left(x\right)=r.x+b \end{array}$$

(16b) $$\begin{array}{l} J\left(r,\xi \right)=\frac{1}{2}∥r{∥}^{2}+C\overset{z}{\underset{n=1}{\sum }}\xi_{n} \end{array}$$

(16c) $$\begin{array}{l} \left(x_{n}.r+b\right)\geq 1-\xi_{n}\text{ for }y_{n}=+1 \end{array}$$

(16d) $$\begin{array}{l} \left(x_{n}.r+b\right)\geq 1+\xi_{n}\text{ for }y_{n}=-1 \end{array}$$

(16e) $$\begin{array}{l} \xi_{n}\geq 0\forall n \end{array}$$

where ∥ *r* ∥^2^ = *r*^*T*^*r*, C is positive regularization parameter, ξ_*n*_ training error measuring parameter, *z* misclassified samples, and *y*_*n*_ is the class labels.

##### 3.4.3.2. Application

Hortal et al. (2016) used an SVM classifier with a radial base function that reduces the run-time and makes it feasible for real-time implementation. The classification accuracy obtained for SVM was relatively higher (75%) compared to other classifiers for fNIRS-based gait rehabilitation (Khan R. A. et al., 2018). SVM, along with the genetic algorithm, are getting their popularity due to enhancement in accuracy for fNIRS signals (Noori et al., 2017). The genetic algorithm (GA) was used to select the optimal feature and then find the SVM model's hyper-parameters. Li et al. (2020a) used a 2-layer-GA-SVM model instead of a single layer to identify four types of self-regulation intentions. The results indicated that the 2-layer-GA-SVM model's accuracy is increased by 13.8% relative to the single GA-SVM model, indicating significant improvements in detecting self-regulated intention using inter-subject BCIs. SVM performance was be studied in several EEG and fNIRS studies to enhance classification accuracy (Mihara et al., 2012; Naseer et al., 2014; Hedian et al., 2018; Kim et al., 2019). SVM with multiple kernels is also getting favor to use in many studies because it quickly expands linear decision boundary into non-linear. The performance of the kernel-based SVM classifier is greatly affected by choice of kernel and its hyper-parameters. Multiple kernels learning with SVM outperformed single kernel SVM classifiers in terms of accuracy and feature fusion problems, especially in gait states classification (Li et al., 2014; Zhang et al., 2017). Ge et al. (2017) used SVM to combine features extracted from EEG-fNIRS signals to achieve an average accuracy of 81.2% for an imaginary motor task. Similarly, Abtahi et al. (2020) used SVM to differentiate datasets for classification between Parkinson's disease and the neurological participant's group. Among datasets, fused EEG-fNIRS achieved the highest classification accuracy compared to individual fNIRS and EEG datasets. For other fused EEG-fNIRS applications, SVM yields an effective classification accuracy (Aghajani et al., 2017; Li et al., 2017).

#### 3.4.4. Gradient Boost Decision Tree (GBDT)

GBDT proposed by (Friedman, 2001) is suitable for the intention detection model in real-time and handle large scale data (Li et al., 2020b). The gradient boosting process involves three components: (1) loss function, which needs to be optimized; (2) weak learner for making a prediction; and (3) additive model, which is used to add weak learners to minimize the loss function. The loss function is dependent upon the nature of the problem. Decision trees (specifically regression trees) are used as the weak learner in gradient boosting. The additive model connects trees to model (Zheng et al., 2017). Therefore, by continually adjusting and optimizing the weak learner's weight to make it a keen learner, the loss function can be minimized and optimized.

#### 3.4.5. Random Forest (RF)

The core concept behind the random forest (RF) is that it randomly selects a subset of available features in feature space and train decision tree classifiers based on these random vectors. RF repeats the process with many of such random features subsets to generate many decision trees (Breiman, 2001). The final output is the fusion of all other outputs of all decision trees. The algorithm is less sensitive to the curse of dimensionality and sufficient for both fNIRS and EEG application, even with less training data (Steyrl et al., 2016; Liu D. et al., 2017; Liu et al., 2018; Wang et al., 2019).

#### 3.4.6. K-Nearest Neighbor (KNN)

This technique's objective is to allocate an unseen point for a dominant class between its k nearest neighbors points within the training set (Lotte et al., 2007). For a significantly high value of k and sufficient training points, KNN can estimate any function to draw a non-linear decision boundary. The function can be Euclidean distance or Mahalanobis distance. KNN is not a very accepted algorithm for BCI application due to its sensitivity toward the curse of dimensionality (Friedman, 1997). However, with low dimensional features, it proved efficient (Borisoff et al., 2004; Khan R. A. et al., 2018).

#### 3.4.7. Deep Neural Networks (DNNs)

Till 2017, methods of DDNs do not show any significant improvements compared to state-of-the-art techniques used for the classification of bio-signals in BCI (Lotte et al., 2018). However, recent research shows its future potential due to its ability to learn useful features and classifiers from raw data simultaneously. Ghonchi et al. (2020) used a combination of convolutional (extracting spatial features) and recurrent neural networks (extracting temporal features) to achieve an accuracy of 99.63% with the proposed model. Tortora et al. (2020) used LSTM deep neural network to differentiate between swing and stance states for both individuals and combine leg movements. Similarly, spatiospectral representation learning (DNN topology) is used to differentiate between four walking conditions using EEG signals (Goh et al., 2018). A few other research show increase in classification accuracy for fNIRS (Ho et al., 2019) and EEG signals (Zeng et al., 2018) using DNNs. Chiarelli et al. (2018) found a significant increase in classification accuracy for multimodel EEG-fNIRS recording than standalone EEG and fNIRS signals and other classification algorithms. Sirpal et al. (2019) proposed a deep recurrent neural network for seizure detection in multimodel EEG-fNIRS recording and found that this promising framework can be used in future EEG-fNIRS models to make detection and prediction.

### 3.5. Gait Applications

EEG and fNIRS are used for a wide range of gait applications. However, a few popular applications of gait are discussed in this section.

#### 3.5.1. Balance Control

Although the articles reviewed in this study do not have much focus on balance control. However, the core importance of neuro-imaging techniques, especially EEG and fNIRS, used to investigate the underlying neural and hemodynamic changes during static and dynamic balance control in humans cannot be ignored. Wittenberg et al. (2017) reviewed neuro-imaging techniques to investigate the cognitive, sensory, and mechanical challenges of static and dynamic balance control. Only a few studies used multi-imaging techniques in investigating balance control. Al-Yahya et al. (2016) used fNIRS and fMRI to find prefrontal activation in both single-task and dual-task conditions and their relation with gait measure. fNIRS data were acquired during treadmill walk while fMRI data are recorded during simulated walking. Enhancement in brain activity changes was found in dual-task conditions compared to single task. Current challenges in balance control are the development of validation for multi-imaging modalities, especially in non-portable neuro-imaging techniques such as fNIRS. Although fMRI has a superior spatial resolution compared to fNIRS, fNIRS hardware mobility offers the advantage of studying the full range of balance challenges. Therefore, future research should investigate models linking EEG and fNIRS. Researchers working to improve neuro-imaging hardware and software should focus on technical challenges to combine fNIRS and EEG modalities. The multimodal mobile fNIRS and EEG system can affect spatial and temporal resolution, providing additional brain activity insights involved in balance control tasks. Currently, only a few studies used mobile EEG (Bulea et al., 2015; Kline et al., 2015; Beurskens et al., 2016; Nathan and Contreras-Vidal, 2016; Oliveira et al., 2016) and fNIRS (Lu et al., 2015; Takeuchi et al., 2016) modalities for human balance control investigation. Beurskens et al. (2016) found decreased alpha (EEG) activity during cognitive dual tasking. Bulea et al. (2015) investigated the balance challenge by performing a steady-state walk on a treadmill. Two fNIRS studies investigated dynamic balance control during overground walking (Lu et al., 2015; Takeuchi et al., 2016).

#### 3.5.2. Gait Intention Detection

The development of real-time BCI-based gait intention is essential, particularly in designing useful assistive and rehabilitation devices. Among many other significant issues in detecting BCI-based intention is external noise, especially for real-time conditions and classification accuracy. Currently, only a few BCI-based systems are developed for online classification for gait intention detection and its implemented to exoskeletons used for lower limb gait rehabilitation. EEG signals are widely used in detecting gait cycles such as start and stop (Sburlea et al., 2015; Hortal et al., 2016), sitting, and standing intentions (Bulea et al., 2014) before movement execution. In other applications, EEG signals are used to trigger robotic devices by continuous classification and asynchronous detection of lower limb movement (Liu et al., 2018). A pseudo-online BCI system to detect the unexpected obstacle was developed with an average accuracy of 63.9%, which can help its implementation in real-time BCI systems. Future works can help increase the accuracy of such a BCI system to make them more feasible for real-time applications (Elvira et al., 2019). A similar EEG-based study was performed to detect the sudden appearance of obstacles for the lower limb exoskeleton during walking with an average accuracy achieved 79.5% (Salazar-Varas et al., 2015). Likewise, hemodynamic changes can also help to detect movement intentions. Li et al. (2020b) performed an fNIRS-based study to detect self-paced walking intention, which forms a foundation for the fNIRS-based BCI system for control of gait assistive devices. Li et al. (2020a) proved the feasibility of the fNIRS-based BCI system for decoding and detecting the motion intention in dynamic situations. It comprehends the potential for practical application of the fNIRS-based BCI system in controlling gait-related assistive devices. Another fNIRS study detects the motion intention using two variables, i.e., step length and walking speed. It also laid the foundation for classification motion intention under a typical environment to control assistive walking devices in severe motor dysfunction patients (Hedian et al., 2018). Assistive tools for the patients can be gradually removed to increase cognitive involvement in the process (Costa-Garciacutea et al., 2019).

#### 3.5.3. Parkinson's Disease (PD)

PD is a specific disease-causing gait and balance disorder (Schoneburg et al., 2013; Galna et al., 2015). EEG and fNIRS are widely used for investigating cortical activation duration walking and balancing task for PD patients (Stuart et al., 2018). PD patients find it difficult to perform any secondary task during walking; the fNIRS device is proved to be feasible to observe the pre-frontal activation during dual-tasking (Nieuwhof et al., 2016) and help with rehabilitation. Stuart et al. (2018) found that many studies use fNIRS rather than EEG to observe the pre-frontal activation in PD patients. However, hybrid EEG-fNIRS can help us better understand cause and effects during the rehabilitation of PD patients as it gives us both the neuronal and hemodynamics information simultaneously.

#### 3.5.4. Rehabilitation

Due to portability and excellent temporal and spatial resolution of both EEG and fNIRS helping patients during gait rehabilitation in terms of wearable lower limb exoskeletons, orthosis, prosthesis, and other assistive robotic devices (Belda-Lois et al., 2011; Chéron et al., 2012; Castermans et al., 2013; Tariq et al., 2018; Hobbs and Artemiadis, 2020). Belda-Lois et al. (2011) reviewed gait therapies used in gait rehabilitation comprise classical gait rehabilitation techniques, FES, BCI systems, and assistive robotic devices. There is not enough evidence regarding classical gait regeneration techniques to conclude that one method is more effective at improving gait than another. The combination of different rehabilitation techniques seems to be more effective than excessive gait training alone. Robotic devices require further research to demonstrate their suitability for training their effects on real-time over the ground walk. Non-invasive BCIs are limited to upper limb rehabilitation. However, some recent works suggest that there may be a standard mechanism that can contribute to the rehabilitation of both the upper and lower limb. Advancement in EEG and fNIRS enables researchers to detect signals from specific cortex regions during motor tasks to develop future BCIs. Future research will analyze the impact of rehabilitation on brain plasticity, align treatment resources to meet each patient's needs, and optimize the recovery process. EEG-based robot-assisted gait rehabilitation is useful to promote mobility in stroke patients (Calabrò et al., 2018). EEG-based neural decoding helps to design a patient-centered closed-loop EEG-based BCI system for better rehabilitation of lower limb and enhance cortical plasticity (Contreras-Vidal et al., 2018). The system can be further improved for rehabilitation (Do et al., 2013). Fused EEG-fNIRS can help find spatial and temporal information changes in cortical activation patterns to understand better robot-assisted gait rehabilitation (Berger et al., 2019). Clinical deployment of the classifier could be a significant step to real-time BCI rehabilitation. Appropriate post-processing steps can be applied to enhance accuracy and reduce the time (Bulea et al., 2014). In some BCI-based rehabilitation studies, it was concluded from the feedback by the subjects the comfortability of suspension and body fixation should be improved considering the situation of disabled patients (Gui et al., 2017). Future work should focus on improving gait rehabilitation efficacy and conducting long-term clinical experiments on paraplegic patients.

#### 3.5.5. Non-invasive Brain Stimulation (NIBS)

NIBS techniques are widely used in healthy adults to investigate brain mechanisms or modify and enhance cognitive, behavioral, social, and emotional processes (Finisguerra et al., 2019). NIBS is broadly classified into transcranial magnetic stimulation (TMS) and FES (Liew et al., 2014). FES is further classified into three major categories: transcranial alternating current stimulation (tACS), transcranial direct current stimulation (tDCA), and transcranial random noise stimulation (tRNS). The future aspect of fused EEG-fNIRS could be the feedback cortical activation pattern measurement to identify regions in NIBS (Teo et al., 2016; Berger et al., 2018). FES can be set up as portable and wireless systems, thus having complementary capabilities as well as EEG and fNIRS (McKendrick et al., 2015). For example, it can help identify hypo-/hyperactivity and gait disorders to determine and guide brain stimulation protocols. It can be applied during robot-assisted gait rehabilitation to modulate neural networks that support gait rehabilitation (Teo et al., 2016). The use of FES combined with different walking techniques was shown to lead to improvements in hemiplegic gait. Hong and Khan (2017) suggested that hybrid brain signal acquisition electrical stimulation can improve the brain recovery process, especially for stroke patients. For FES, the correct brain region is essential; hence, integrating neuronal and hemodynamics signals can better localize it.

## 4. Discussion and Future Prospect

Fused EEG-fNIRS can help in understanding the neurophysiological mechanisms underlying motor behavior and gait impairments due to neurological diseases (Berger et al., 2019). Both EEG and fNIRS are non-invasive, portable, and cost-effective brain monitoring modalities. Furthermore, EEG and fNIRS are suitable modalities for real-time clinical applications involving gait analysis. Since fused EEG-fNIRS captures spatial and bio-electrical temporal brain signal changes, new features related to brain activation and connectivity can be extracted. Understanding and identifying such new features during a complex gait process will be a step forward in the field of hBCI-based gait analysis. However, many questions remain still unanswered, such as how both these bio-electrical and hemodynamic signals are related? How can the fusion of both signals provide benefit in terms of investigating gait disorders caused by brain dysfunction? Some of the key findings from different studies documenting the advantage of fusing EEG-fNIRS are as follows:

1. The relation between neuronal changes and neuro-vascular coupling needs further investigation. Lachert et al. (2017) found that during the finger-tapping task, HbO increases along with a decrease in HbR concentration and amplitudes of alpha and beta EEG rhythms. A decrease in HbO concentration in the primary motor and somatosensory cortex area with an increase in EEG alpha power following 10 Hz and 20 Hz transcranial tDCS was observed. The authors report that reduced alpha and beta oscillations in the cortical motor network are expected to be accompanied by an increase in HbO, which is a finding that is supported by related literature investigating neural correlations during gait.
2. Fused EEG-fNIRS provides detailed spatiotemporal information of neuro-physiological changes, both while performing a task and during rest state. Simultaneous measurements of EEG and fNIRS can improve the classification accuracy by combining the feature space of these two modalities (Leamy et al., 2011; Fazli et al., 2012; Buccino et al., 2016; Ge et al., 2017; Li et al., 2017).
3. It is possible to use data acquired from one type of modality to remove artifacts from other types^1^. Today the primary focus of multimodal integration of EEG-fNIRS is to enhance the performance of hBCI for MI tasks. Some of the studies already demonstrated performance gains by fusing EEG-fNIRS in MI tasks (Khan et al., 2014; Buccino et al., 2016), which is of utmost importance to many gait applications such as rehabilitation and gait intention detection.

Although many studies report that fused EEG-fNIRS BCI systems yield superior performance compared to single EEG and single fNIRS-based BCI systems (Khan et al., 2014; Koo et al., 2015; Naseer and Hong, 2015; Ahn and Jun, 2017), there is still a lot of research that needs to be conducted to understand the different aspects of fusing EEG and fNIRS fully. From a broader perspective, the first problem is related to the hardware and instrumentation used to collect both signals using a single device. The second problem relates to the nature of the signals themselves from two different domains (temporal and spatial) that need to be jointly processed (Ahn and Jun, 2017). Another problem that is encountered in the case of gait analysis is the motion artifact that arises due to movements, instrumental, and external light interference (Vitorio et al., 2017). Some of the below problems must be resolved to make quick progress toward real-time implementation of the EEG-fNIRS-based hBCI system:

1. Since the fNIRS signal's response is slower than the response of EEG. Researchers are trying to investigate new features and classification algorithms for immediate detection of hemodynamic changes (Buccino et al., 2016; Hong et al., 2018; Khan M. J. et al., 2018). The hemodynamics delays can be estimated with computational and simulation models (Buxton et al., 2004).
2. Temporal synchronization is also a critical problem due to the information transfer rate in hybridization. Few computational methods such Bayesian methods capturing prior information (Morioka et al., 2014) and feature normalization (Ahn et al., 2016) provide solutions for better performance.
3. Recording neural activity from the same location is usually a tedious task. The same channel configuration can be achieved when each EEG electrode is placed between the emitter and detector of the corresponding fNIRS optode. EEG electrodes are comparatively smaller in terms of size relative to fNIRS optodes. The infrared light quantification in fNIRS is negatively affected by dense hair, which not only causes a low signal-to-noise ratio but also poses a problem related to the same channel configuration.
4. Placing a larger number of EEG electrodes and fNIRS optodes for simultaneous brain activity measurement can cause higher dimensionality and higher computational costs. Some commonly used spatial filtering methods of common spatial patterns can reduce dimensionality and allow us for more useful information. But, the number of electrodes and optodes should still be carefully considered for experimentation.
5. Most of the studies in hBCI involve only healthy subjects. Before adopting hBCI for patients instead of healthy subjects, more research is required. Furthermore, the comfort of EEG-fNIRS hBCI need to be enhanced to be adopted by patients that require a high level of comfort. Although many studies that document high accuracy for healthy subjects suggest it is possible to generalize those results for patients, the reality may differ (Chaudhary et al., 2017).
6. In real scenarios where conditions differ from lab-controlled environments, there is a need for more progress in the design of reliable and ergonomic hardware. The recent development in custom-made wireless and compact EEG-fNIRS can help to solve these issues (von Lühmann et al., 2015).

Hybridization of EEG and fNIRS can provide promising results for gait application. Some useful recommendations are deduced from the literature that can help the researchers to better plan gait studies. In most fNIRS studies, there is no standardization of experimental protocols. Because of this lack of standardization, it is recommended to report all technical information such as source-detector separation, sampling frequency, the total number of channels, differential path length factor (DPF) values, assessment methodology (with resting and task time), etc. Due to portability limitation in most of the existing EEG and fNIRS devices, the experiments are usually performed in lab-controlled environments. However, the recent hardware development in hybrid EEG-fNIRS devices solves this portability issue allowing mobility even in an uncontrolled environment. Before starting any gait assessment, it is recommended to carefully consider hardware specifications and characteristics in terms of portability, the number of channels (electrode and optodes), sampling frequency, amplifier, sensitivity, noise level, the capacity of battery backup, and continuous recording (for portable devices), and range of wireless digital transmission. Additional sensory devices for recording non-brain physiological signals such as heart rate, blood pressure, skin conductance, and respiration can help denoise brain signals. Regions of interest (ROIs) should be carefully taken into consideration when selecting the hardware. In various walking and balance studies, ROIs appeared to be selected based upon the hardware limitation rather than task-specific regions (Stuart et al., 2018). Hong et al. (2018) summarized different algorithms that could be useful to determine ROIs (see section 3.2.8). Most of the gait studies recommend using 10-20 or 10-10 international positioning systems for optode/electrode placement. Increasing the number of channels may allow access to many different ROIs, but it also increases the computational cost. In the case of some fNIRS devices, an increase in the number of channels reduces the sampling frequency. Hence, the number of channels should be carefully selected. During the experimental paradigm design, the duration of the baseline time, the in-between rest time, and task time should also be considered carefully. There is no commonly used standard baseline time in the fNIRS studies yet. However, a baseline rest of a minimum of 30 s is recommended (Herold et al., 2018). Longer baseline time may affect the fNIRS recording as it is sensitive to mind wandering (Durantin et al., 2015). Constant DFP factor value should be avoided because of its dependency upon the age of the participant, wavelengths, and source-detector separations. In this case, it is recommended to take into consideration the age and wavelength values for computing DFP instead of relying on the default values. Some open-source toolbox such as “nirsLAB” can help in selecting DPF value accordingly.

Common sources of noise in gait assessment could be due to motion artifacts, instrumental noise, and physiological noise. Motion-related artifacts can be removed using the filtration methods discussed in section 3.2. Some other filters, such as task-related component analysis, Kalman filter, and hybrid filter techniques combining, for instance, spline interpolation with Savitzky-Golay filtration, are also recommended to remove motion artifacts in fNIRS signal (Tanaka et al., 2013; Jahani et al., 2018). Physiological noise can be removed by using low- and high-pass filters. This type of noise can also be removed by recording the physiological parameter with additional pieces of equipment. Open-source toolboxes could help to quickly analyze fNIRS data such as “NIRS Brain AnalyzIR” (Santosa et al., 2018) or “HOMER” (Huppert et al., 2009). Baseline correction and averaging across the channels are usually performed after the filtration of the signals.

For acceptable classification accuracy, the identification of prominent features is essential. In hybrid EEG-fNIRS analysis, we can classify the features as temporal, spatial, and spatiotemporal. EEG and fNIRS studies' most commonly used features are signal peal, slope, mean, kurtosis, skewness, and power spectrum density. In many gait application, event-related synchronization and desynchronization-based features are combined with the fNIRS feature to improve accuracy. Some other methods already discussed in section 3.3 could also improve the performance. The most commonly established classification algorithms in hybrid EEG-fNIRS studies for gait are already discussed in section 3.4. However, there are other algorithms that are not discussed in this study, such as extreme learning machines and vector phase analysis that are used in other than gait applications involving hybrid EEG-fNIRS (Hong et al., 2018). In many of the gait and balance studies, cortical activation associated with postural change was reported. These activation types could be useful for investigating gait disorders and controlling robotic interfaces, especially for rehabilitation purposes.

## 5. Conclusion

The increase in the number of balance and gait disorders in young and older adults is becoming a real challenge and burden on the health sector. Today, the fusion of different brain and non-brain signals help medical doctors, physicians, and researchers better investigate various gait challenges. A combination of hemodynamical (fNIRS) and electrophysiological (EEG) modalities to form a hybrid BCI (hBCI) is a novel methodology for further enhancement in the performance of BCI in terms of classification accuracy, increase of the number of control commands, and decrease in the response time of BCI. The review summarizes the potential of the EEG-fNIRS-based hBCI systems for investigating gait and balance disorders. The EEG-fNIRS-based hBCI for the lower extremity remains still an under-investigated research axis that holds great potentials for a breakthrough in the field of designing BCI for gait applications.

## Author Contributions

HK and PM conceived the idea. HK conducted the review and drafted the initial manuscript. PM, AY, and NN, who oversaw the project, structured the manuscript, and guided it to completion. HH, AY, and PE contributed to design the study, drafting, reviewing, and implications of the manuscript. All authors approved the submitted version of the manuscript.

## Conflict of Interest

The authors declare that the research was conducted in the absence of any commercial or financial relationships that could be construed as a potential conflict of interest.

## Footnotes

^1^Likewise, EOG modality was used to remove eye-movement artifacts from EEG data (Liu et al., 2018).
